# It Is Dangerous to Go Alone: Strategies to Optimize PET Biocatalysis and Upcycling through Enzymatic Synergism

**DOI:** 10.1021/acsomega.5c02068

**Published:** 2025-10-01

**Authors:** Bruno Rampanelli Dahmer, Jeferson Camargo de Lima, José Fernando Ruggiero Bachega, Troy Wymore, Luis Fernando Saraiva Macedo Timmers

**Affiliations:** † Graduate Program in Biotechnology, 186081 Universidade do Vale do Taquari − Univates, Lajeado 95900-000, RS Brazil; ‡ Department of Pharmacosciences, Universidade Federal de Ciências da Saúde de Porto Alegre, Porto Alegre 90050-170, RS Brazil; § Department of Chemistry, 6572 University of Pennsylvania, Philadelphia, Pennsylvania 19104-6243, United States; ∥ Graduate Program in Medical Sciences, Universidade do Vale do Taquari − Univates, Lajeado 95900-000, RS Brazil

## Abstract

Current mainstream methods of plastic recycling are inadequate, producing lower-quality polymers, or are cost-inefficient, requiring expensive reagents operating under harsh conditions. Enzymatic biodegradation of poly­(ethylene terephthalate) (PET) plastic was first extensively described in 2005, and several PET degrading enzymes have been identified since then. Recently discovered or developed PET-degrading enzymes are inhibited by the intermediate reaction product, mono­(2-hydroxyethyl) terephthalate (MHET). Therefore, the enzymatic conversion of PET into its original components, terephthalic acid (TPA) and ethylene glycol (EG), is still inefficient. The synergistic cooperation between PET hydrolases and enzymes capable of hydrolyzing released intermediate products can increase reaction efficiency, reduce separation costs, and enable the full recovery of the basic components of PET for their potential conversion into value-added chemicals. This review aims to provide an overview of enzymes capable of degrading intermediate products and potentially solving the heterogeneous product solution problem, their structures and activities, importance, and potential applications in combination with PET hydrolases.

## Graphical abstract

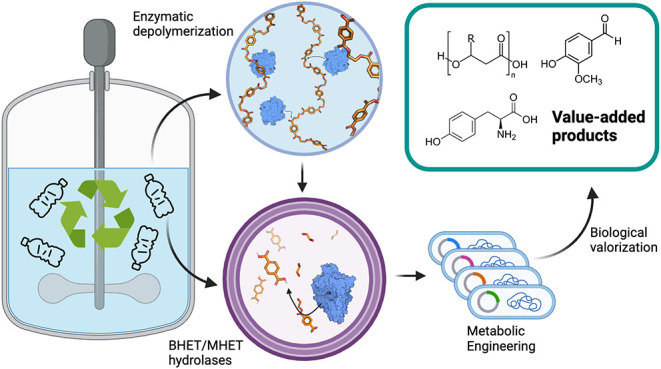

## Introduction

Plastics are virtually integral to modern society due to their functional properties, biochemical inertness, low production cost, and broad range of applications. However, their extensive use has led to significant environmental challenges, fueling a sustainability crisis. Although the large-scale industrial production of plastics only started in the 1950s, it has been estimated that around 7800 Mt of polymer resins and fibers have been produced since then.^1^ The packaging sector comprises almost half of the total market demand for plastics, where polyethylene terephthalate (PET) finds one of its main applications in single use, disposable food and beverage packaging.^1,2^ The very properties that enabled the widespread adoption of synthetic polymers in lieu of other materials are also responsible for plastics becoming a critical environmental problem. Landfills are still the most common destination given to postconsumer plastics, some of which may erode over a long period of time and release microparticles into the environment. Traditional recycling methods, primarily mechanical and chemical, have limitations, including quality degradation and high energy consumption.^3,4^

Conversely, biological recycling of plastic waste operates under less severe conditions of temperature and pressure, making it a promising alternative for sustainable plastic waste management. Biocatalytic conversion depolymerizes the plastic substrates in monomers and oligomers that could be used as feedstock for recycling or production of new plastics, or the synthesis of added-value substances.^5^

The enzymatic biodegradation of synthetic plastics is dependent on the types of covalent bonds present along the polymeric chains and the abundance of similar, specific bonds found in natural polymeric substrates. Plastics that contain hydrolyzable bonds, such as polyesters, polyamide oligomers, and ester-based polyurethane, are highly susceptible to hydrolytic attack. Esters are prevalent in natural biopolymers, such as in lipid- and phenol-based barriers on the surface of plant cells, like cutin and suberin.^6^ Likely due to its similarity with naturally occurring biopolymers, the fossil-based polymer PET is the most extensively studied plastic concerning its enzymatic biodegradation. Most of the enzymes capable of degrading PET (and therefore termed PET hydrolases) are promiscuous α/β-hydrolases of the carboxylic ester hydrolases superfamily (EC 3.1.1), including esterases (EC 3.1.1.1), lipases (EC 3.1.1.3), and cutinases (EC 3.1.1.74). *Ideonella sakaiensis* (*Is*), a bacterium capable of utilizing PET as its sole carbon source, produces two enzymes, *Is*PETase (EC 3.1.1.101) and *Is*MHETase (EC 3.1.1.102). These enzymes can break the ester bonds along the polymeric chain, releasing bis­(2-hydroxyethyl)-terephthalate (BHET), mono-(2-hydroxyethyl) terephthalate (MHET), terephthalic acid (TPA) and ethylene glycol (EG) (Figure 1).

**Figure 1:**
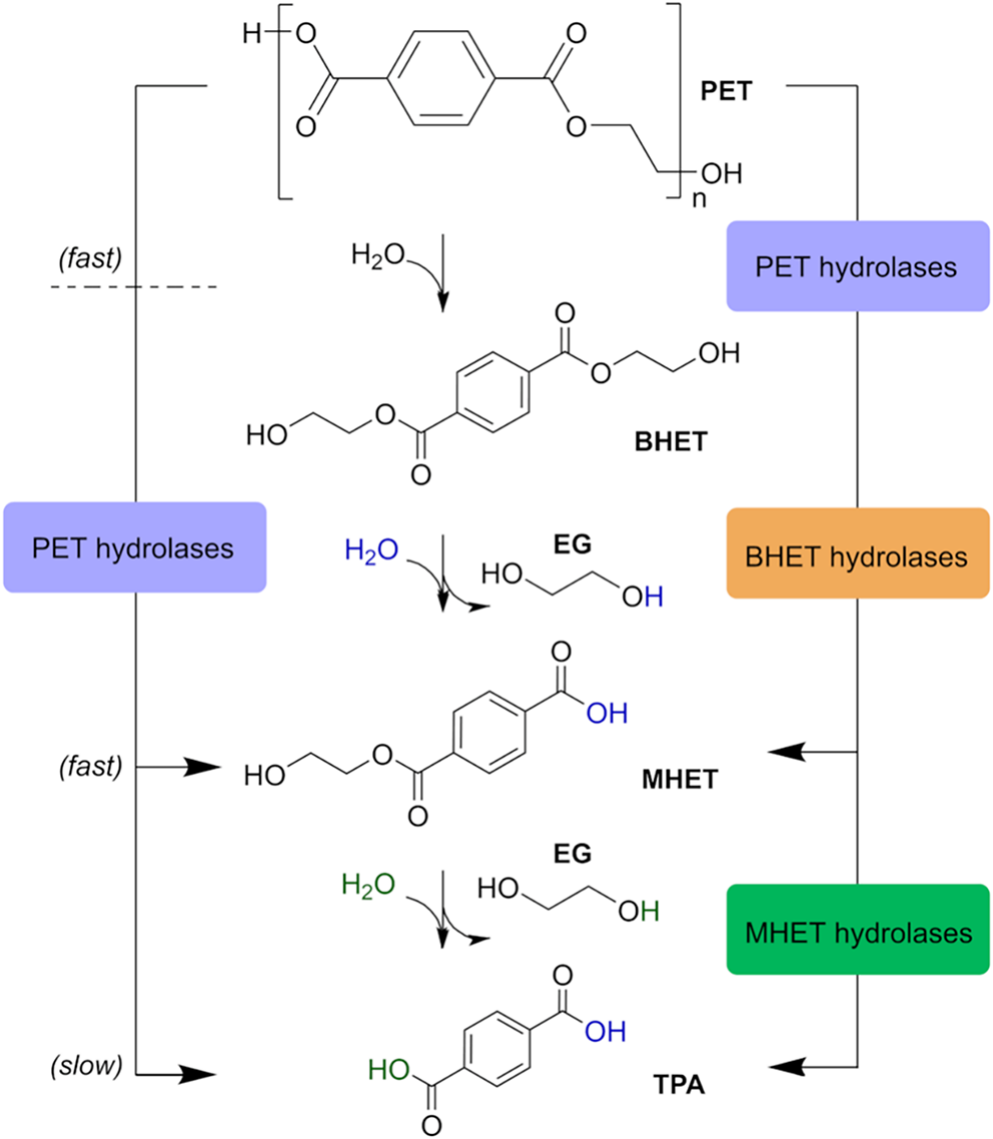
General biocatalysis of PET to TPA and EG through the intermediates BHET and MHET. Generally, some PET hydrolases are capable of fully degrading PET to TPA and EG, but product inhibition or the slower rate of hydrolysis of intermediate products leads to their accumulation in the system. BHET and MHET hydrolytic enzymes can more efficiently break down intermediate products resulting from PET degradation.

PET hydrolases usually possess shallow and open active sites that are able to accommodate bulky polyester chains, such as PET. However, this architecture renders them less efficient at cleaving low-molecular-weight intermediate products (especially MHET) and susceptible to product inhibition, broadly understood here as competitive or nonproductive binding of hydrolysis products.^7^ As such, the heterogeneous products yielded from biocatalytic breakdown of PET plastic represent a bottleneck to PET recondensation or the synthesis of high-value derivatives. Incorporating auxiliary carboxylesterases with a preference for shorter acyl chain esters, such as MHET and BHET, can assist PET hydrolases by accelerating intermediate turnover and can further enable the recycling, upcycling, or full biological conversion of postconsumer PET plastic. Therefore, exploring the biotechnological and industrial applications of the PET enzymatic biodegradation process may benefit from a better understanding of the complementary roles played by PET hydrolases and BHET/MHET-hydrolyzing enzymes.

Thus, this review is centered on exploring the structural and functional properties of several enzymes known to mediate the catalysis of PET-derived oligomers, mainly BHET and MHET, and the potential applications that result from their synergy with PET hydrolases. Additionally, the review explores recent efforts to elucidate and tailor the metabolic pathways of natural or engineered microorganisms for uptake, assimilation, or conversion of PET waste-derived substances into value-added materials.

## BHET and MHET Hydrolytic Enzymes

This section presents a selection of enzymes with reported or proposed activity toward PET hydrolysis intermediates, chosen based on both established biochemical evidence and functional potential inferred from structural or mechanistic traits. While some enzymes included in this review have been formally and directly characterized for their activity toward soluble PET depolymerization intermediates, others (though not originally classified as such) are included based on biochemical characteristics, inferred substrate preference, or literature-reported activity patterns that suggest a promising potential role in intermediate turnover. As such, we selected enzymes that either: (i) have been directly described as MHET/BHET hydrolases, or (ii) exhibit traits consistent with intermediate-specific activity, such as low TPA/MHET yield from PET but comparatively better turnover of short-chain oligoesters. In this section, we seek to highlight some of these enzymes, outlining their defining features. Table 1, at the end of this section, also summarizes and offers a comparative overview of the auxiliary enzymes included in this review.

**Table 1:** Summary and Comparative Overview of Auxiliary BHET/MHET Hydrolytic Enzymes

Enzyme	Activity Toward BHET/MHET	Reaction Temp. (°C)	Notes	Reference
*Ca*LipB	*K*_M_ = 13.3 ± 4.3 mM, *k*_cat_ = 0.89 ± 0.15 s^–1^ (BHET, pH 7);	50–60	Thermostable. Highly promiscuous lipase with broad applications in industry (food, cosmetics, pharmaceutical). pH-dependent regioselectivity: BHET > MHET at pH 7; MHET > BHET at pH 5.	^16,17^
	*K*_M_ = 23.8 ± 8.8 mM, *k*_cat_ = 1.25 ± 0.29 s^–1^ (MHET, pH 5).			
*Ta*Est1	Not quantified.	60–75	Thermostable. Pentamutant (*Ta*Est1_5M) with enhanced activity and substrate range.	^21,24^
*Tf*Ca	Preference for BHET over 2PET; relative *K*_A_ = 0.085 mg/mL, relative *k*_τ_ = 0.35 min^–1^.	50–60	Thermostable. Engineered variant with improved activity. Enhanced MHET and BHET hydrolysis via I69W/V376A variant (2.6-fold and 3.3-fold higher, respectively).	^28,31^
*Tt*CE	Not quantified.	80	Thermophilic. Active across broad temperature ranges and pH levels. Potentially suitable for harsh industrial conditions. Shown to synergistically degrade PET in combination with LCC, with pure TPA and EG yields ∼30–100% higher.	^34,37,75^
*Bs2*Est	*K*_M_ = 8.84 mM, *k*_cat_ = 2.10 × 10^5^ s^–1^ (BHET)	30	Industrially robust source organisms. Generally thermostable.	^45^
	*K*_M_ = 5.10 mM, *k*_cat_ = 6.46 s^–1^ (MHET)			
*Bs*CE	*K*_M_ = 0.77 ± 0.08 mM, *k*_cat_ = 13 ± 0.80 s^–1^ (BHET);	50		^49,76^
	*K*_M_ = 1.3 ± 0.51 mM, *k*_cat_ = 0.056 ± 0.015 s^–1^ (MHET).			
*Bs*Est (PET-86) & *Chry*BHETase (PET-29)	*K*_M_ = 0.1 ± 0.02 mM, *k*_cat_ = 37.7 ± 1.98 s^–1^ (*Bs*Est);	30–60	Both showed high substrate preference for BHET over aliphatic esters. Thermostable variants with up to 3.5-fold improved catalytic efficiency and up to 7.0-fold improvement in TPA production compared to seven benchmark PET hydrolases alone.	^74^
	*K*_M_ = 0.08 ± 0.03 mM, *k*_cat_ = 102.2 ± 5.18 s^–1^ (Δ*Bs*Est);			
	*K*_M_ = 0.09 ± 0.02 mM, *k*_cat_ = 6.85 ± 0.53 s^–1^ (*Chry*BHETase);			
	*K*_M_ = 0.12 ± 0.03 mM, *k*_cat_ = 12.88 ± 1.24 s^–1^ (Δ*Chry*BHETase);			
*Is*MHETase	*K*_M_ = 0.0073 mM, *k*_cat_ = 31 s^–1^ (MHET, WT).	30–40	Canonical MHET hydrolase, but thermolabile above 44 °C.	^55,56^
	*K*_M_ = 1.45 mM, *k*_cat_ = 3.205 s^–1^ (BHET, R411K/S416A/F424I triple mutant).		*Is*MHETase^R411K/S416A/F424I^ variant with BHETase activity.	
Mle046	No detectable activity toward, or inhibition by, BHET.	20–40	Broad temperature range: mesophilic but cold-active enzyme. Marine adapted.	^67^
	For MHET, *K*_M_ = 2.638 ± 0.797 mM, *k*_cat_ = 80.9 ± 15.8 s^–1^.			
*Mar*CE	Not quantified. Predicted through molecular docking with BHET.	30	Structurally similar to *Tf*Ca, with two lid domains. Marine adapted.	^72^
KL-MHETase	*K*_M_ = 4.56 mM, *k*_cat_ = 21.43 s^–1^ (MHET).	50	Computationally designed thermostable enzyme. Tested in a large reaction system (100 g/L), with 90% PET depolymerization when fused to FAST-PETase.	^69^

### *Candida Antarctica* Lipase B (CaLipB)

*Ca*LipB is a highly promiscuous, multipurpose enzyme with various industrial applications and organic synthesis in the food, cosmetic, pharmaceutical, and chemical industries, thanks to its high activity, broad substrate specificity, tolerance to a wide pH range, ability to function in both aqueous and organic environments, and thermal stability.^8–11^ Uppenberg et al.^12,13^ solved the first crystallographic structures of *Ca*LipB. The enzyme is monomeric and belongs to the α/β-hydrolase superfamily, composed by 317 amino acid residues arranged in a core structure comprising a central, mostly parallel, seven-stranded β-sheet surrounded by α-helixes. Its fold exhibits a stable scaffold for the conserved Ser-His-Asp catalytic triad of serine hydrolases; however, the serine hydrolase consensus sequence around the catalytic Ser105 differs in *Ca*LipB, from the GxSxG motif to TWSQG. His244 and Asp187, which are stabilized by an oxyanion hole (Thr40 and Gln106), make up the remaining residues of the catalytic site. The binding site of *Ca*LipB is further divided into two compartments with different affinities: the first accommodates the acyl moiety of esters while the latter binds to alcohols.^12,14^

The presence of a lid domain covering the active site of *Ca*LipB was a contentious topic of discussion due to a lack of structural evidence and of interfacial activation. Stauch et al.^15^ reported crystallographic structures of *Ca*LipB in open and closed conformations, showing that α-helix_5_ and α-helix_10_ are responsible for the latter through a combination of effects stemming from properties of the substrate, media, and the amino acid composition of the lid region. In its open conformation, α-helix_5_ shows a series of aliphatic residues along the channel leading to the active site of *Ca*LipB; in the closing of the binding site, α-helix_5_ undergoes a conformational change and unfolds into an unstructured loop resulting in a salt bridge between Asp145 and Lys290, which brings in α-helix_10_ and closes the active site, thus hindering substrate accessibility and preventing catalysis.

There is no definite consensus regarding its activity against PET polymer. Evidence for the involvement of *Ca*LipB in PET biocatalysis was initially described by Carniel et al.,^16^ when the authors screened 10 commercial lipases using BHET as a model substrate. Both the BHETase and MHETase functions of *Ca*LipB were identified in synergic combination with the PET hydrolytic activity of *Humicola insolens* cutinase (*Hi*Cut). The combination of these two enzymes resulted in a 7.7-fold increase in TPA yield from PET after 14 days of reaction at 50 or 60 °C.

Initial screenings revealed very little hydrolytic activity of *Ca*LipB toward pretreated PET. However, it displays notable dual BHETase and MHETase functions. While Carniel et al.^16^ evaluated the hydrolysis of MHET by *Ca*LipB at neutral pH, Świderek et al.^17^ employed hybrid quantum chemical/molecular mechanics (QC/MM) molecular dynamics simulations, complemented by experimental Michaelis–Menten kinetics, to explore how the pH-dependent ionization state of *Ca*LipB significantly influences the binding and subsequent hydrolysis of either substrate: *Ca*LipB actually hydrolyzes BHET more efficiently at pH 7 (*K*_M_ = 13.3 ± 4.3 mM, *k*_cat_ = 0.89 ± 0.15 s^–1^) compared to the slower hydrolysis of MHET (*K*_M_ = 15.1 ± 3.2 mM, *k*_cat_ = 0.14 ± 0.011 s^–1^), while it shows a higher preference the latter at pH 5 (*K*_M_ = 23.8 ± 8.8 mM, *k*_cat_ = 1.25 ± 0.29 s^–1^ for MHET, *K*_M_ = 22.5 ± 9.6 mM, *k*_cat_ = 0.52 ± 0.07 s^–1^ for BHET). Hence, under acidic conditions, the enzyme predominantly yields TPA. Conversely, alkaline conditions favor the accumulation of MHET due to the selective hydrolysis of only one ester linkage in BHET. Under acidic conditions, the ionization states and protonation of several *Ca*LipB residues form a neutral hydrogen bond network that enables the binding of both BHET and MHET, allowing for the double hydrolysis of BHET to yield two molecules of EG and one TPA. At pH values above 7, these ionization states and some conformational plasticity of the active site (particularly key distances such as Ser105-His224 and Ser105-Asp134, which can compromise the proton transfer required to activate Ser105 for hydrolysis) destabilize the *Ca*LipB:MHET complex and explain the observed selectivity of the enzyme.

The promising experimental behavior, robustness, and flexible selectivity of *Ca*LipB on top of its extensive commercial and industrial applications may allow for the development of engineered variants with enhanced activity regarding the enzymatic catalysis of PET degradation intermediates.

### *Thermobifida alba* AHK119 Esterase 1 (TaEst1)

The genus *Thermobifida* typically contains two tandem cutinase genes.^18^ *Thermobifida alba* produces two distinct cutinases from different genes, designated as est1 and est119.^19^ Est119, which has been reclassified as cutinase 2, was initially characterized as a polyester-hydrolyzing esterase from *T. alba*.^20^ More recently, it has been demonstrated that Est1 also possesses the ability to degrade ester-type plastics.^21^ Although the two cutinases from *T. alba* exhibit different activities and thermostabilities, they share 95% identity and 98% similarity. Notably, Est1 displays approximately twice the activity compared to Est119.

*Ta*Est1 is an enzyme derived from the thermophilic actinomycete *T. alba*, commonly found in compost environments. *Ta*Est1 is notable for its ability to hydrolyze a wide range of esters and polyesters, making it crucial for polymer degradation and chemical synthesis. Its thermal stability, with an optimal activity range of 60 to 75 °C, suggests it could be valuable in industrial processes, particularly for recycling plastics and producing chemical intermediates.^21^

Although crystallization studies of *Ta*Est1 have been reported,^22^ the crystal structure has not been deposited in the PDB. Consequently, structural details are based on the AlphaFold DB model (AF ID: D4Q9N1). *Ta*Est1 adopts a typical α/β-hydrolase fold, featuring 12 α-helices and 9 β-strands arranged into two homodimers. This fold includes a central β-sheet surrounded by α-helices, which provides structural stability and functionality.^23^ The enzyme’s active site contains a catalytic triad of Ser165, Asp211, and His243, essential for hydrolyzing substrates.

Efforts to optimize the activity and stability of *Ta*Est1 included rational engineering through site-directed mutagenesis, targeting residues near to catalytic triad and regions associated with substrate interaction. To enhance thermostability and catalytic performance, Est1 mutants were engineered, yielding notable results. The Est1 (A68 V/T253P) double mutant (Est1DM) exhibited higher enzymatic activity than both the wild-type Est1 and Est119, maintaining over 70% of its activity after 30 min and over 50% after 60 min at 338 K. Est1DM also demonstrated a broad substrate specificity, effectively degrading both aliphatic and aliphatic-*co*-aromatic polyesters, including PET film.^19^ Further mutation screening identified the Est1_5 M variant (mutations: N213M, T215P, S115P, Q93A, L91W), which achieved a remarkable 90.84% degradation of commercial PET bottles within 72 h—a 65-fold increase over the wild type.^24^ These findings underscore the potential of Est1 mutants in developing efficient enzymes for PET degradation and addressing plastic pollution.

### *Thermobifida fusca* KW3 Carboxylesterase (TfCa)

Hydrolases of both natural and synthetic polyesters have been described in several moderate thermophilic actinomycetes.^25^ Members of the *Thermobifida* genus are frequently isolated as habitants in compost and are major degraders of plant cell wall.^26^ When grown on PET fibers-supplemented media, *T. fusca* produces a carboxylesterase (*Tf*Ca) comprising approximately 497 amino acid residues with a molecular mass of 50 kDa.^27^ *Tf*Ca is a monomeric enzyme build from three domains: a central domain that adopts the canonical α/β hydrolase fold with a central β-sheet surrounded by α-helices, and two lid domains (Figure 2A).^28^

**Figure 2:**
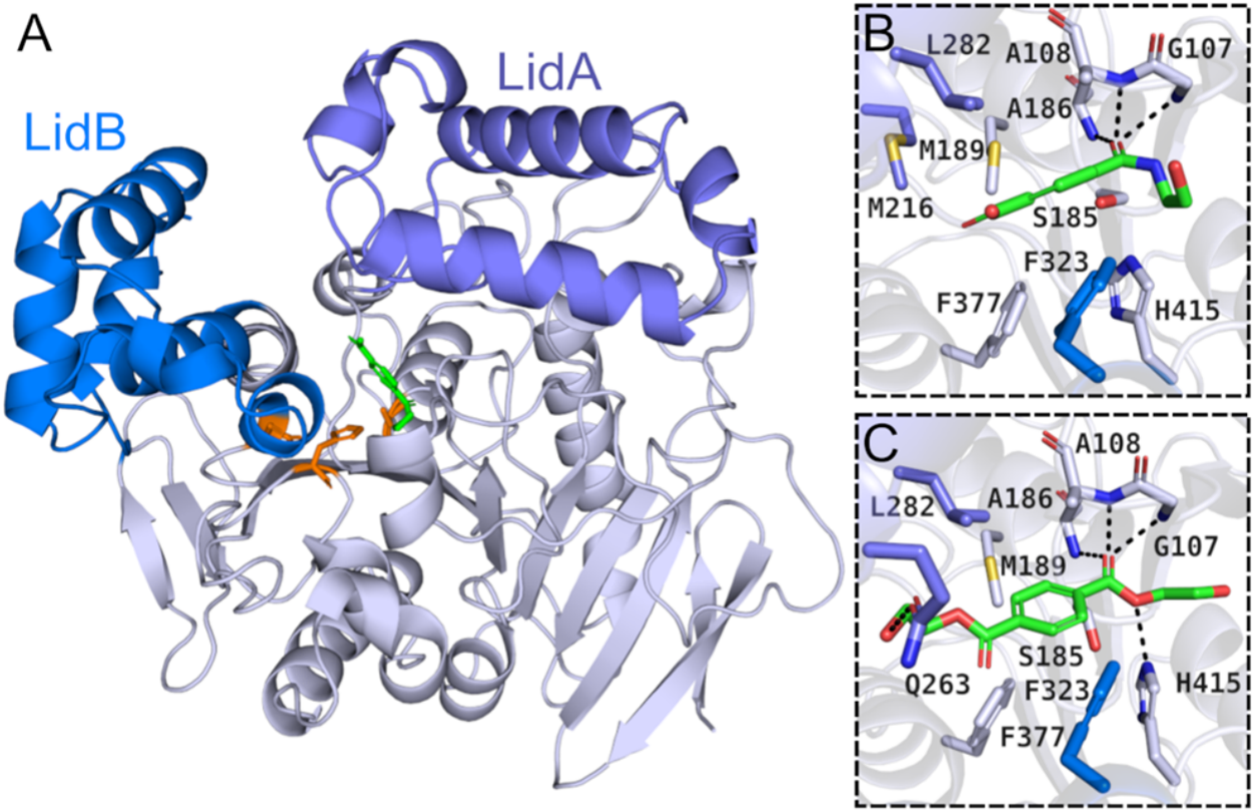
Structure of *Tf*Ca. (A) Crystallographic structure of *Tf*Ca showing its hydrolase domain (white) and its two lid domains (LidA and LidB, purple and blue, respectively); (B) Binding mode of MHETA (green) in the active site of *Tf*Ca; (C) Binding mode of BHET (green) in the active site of *Tf*Ca.

Unlike lipases, which preferentially hydrolyze water-insoluble long-chain triglycerides, carboxylesterases typically cleave short-chain acylglycerols and *p*-nitrophenyl esters.^29^ Consequently, *Tf*Ca has been shown to exhibit catalytic activity against short and medium-chain esters like PET oligomers and intermediate products, being capable of degrading cyclic PET trimers at an optimum temperature of 60 °C with a *K*_m_ of 0.5 mM.^27,30^ Belisário-Ferrari et al.^31^ developed a turbidimetric assay to analyze the enzymatic hydrolysis of PET model substrates (2PET and BHET) and validated it with the hydrolysis reaction carried out by *Tf*Ca. The enzyme showed a clear preference for the less bulky esters in intermediate products, as *Tf*Ca exhibited a 3.5-fold higher *k*_cat_ and 2-fold higher *K*_M_ toward BHET than 2PET.

A variant of *Tf*Ca has been developed by von Haugwitz et al.^28^ through structure-guided rational engineering of residues located in the binding site of the enzyme. Fourteen residues within 5 Å of the ligands were substituted with alanine, with R428A and V376A showing higher activity than the wild-type enzyme, while I69A and M189A were completely inactive and were subjected instead to saturation mutagenesis. The I69W mutant showed double the activity of WT *Tf*Ca, likely due to more favorable interactions with the aromatic moiety of the substrate. A combination of these identified mutations led the authors to *Tf*Ca^I69W/V376A^, which had 2.6-fold and 3.3-fold higher hydrolytic activities on MHET and BHET, respectively.

The authors also obtained crystallographic structures of WT *Tf*Ca bound to a nonhydrolyzable MHET analogue (MHETA) containing an amide bond in place of the ester bond in the original molecule, preventing its hydrolysis by estereolytic enzymes, and BHET. The BHET-bound structure was also made inactive via the E319L mutation, enabling its crystallization. In the binding modes of both complexes, the ligands are located at the bottom of a very hydrophobic deep active site cleft (Figure 2B and C). Three hydrogen bonds are formed in the active site of *Tf*Ca with the amide bond and the hydroxyl end of MHETA, while the side chains of phenylalanine residues (Phe323 and Phe377) are involved in stabilizing the aromatic ring in the TPA moiety of the substrate, analogous to the binding mode of the same substrate in *Is*MHETase (Phe495 and Phe415). Molecular dynamics simulations of enzyme-ligand complexes also revealed a higher propensity for MHET to remain in the substrate binding pocket of *Is*MHETase than *Tf*Ca, indicating that the latter indeed has a lower affinity for MHET than *Is*MHETase.^28^

### *Thermus thermophilus* Carboxylesterase (TtCE)

*T. thermophilus* is an obligate aerobic bacterium, originally isolated from a hot spring in Japan, that exhibits optimal growth at high temperatures, specifically between 70 and 80 °C.^32,33^ The carboxylesterase from *T. thermophilus* (TtCE) is a thermophilic enzyme notable for its exceptional thermal stability. It exhibits enzymatic activity over a broad temperature range (19.7–80 °C) and functions effectively in neutral to alkaline pH conditions (4.0–9.3). TtCE can hydrolyze ester bonds in a variety of substrates, showing a preference for medium-chain esters (C10) and high activity on short-chain substrates.^34^ The combination of its exceptional thermal stability and ability to operate across a wide range of temperatures and pH levels makes the TtCE a promising candidate for biotechnological applications, particularly in processes that require enzymes to function under extreme conditions.

To date, no experimentally resolved structure of TtCE is available; however, one study has employed comparative modeling to investigate structural aspects of an esterase from *T. thermophilus* HB27.^35^ However, two predicted models have been deposited in the AlphaFold database (AF ID: AF-A0A7R7TF72-F1-v4, sourced from *T. thermophilus*, and AF-H9ZPJ1-F1-v4, from *T. thermophilus* JL-18). TtCE adopts the classic α/β-hydrolase fold, characterized by 10 parallel β-sheets and 13 α-helices arranged in a barrel-like configuration. The predicted active site contains the canonical Ser-His-Asp catalytic triad (S75, H203, and D175). These structural insights, particularly the presence of the canonical Ser-His-Asp catalytic triad, enhance our understanding of TtCE’s evolutionary adaptations, contributing to the knowledge of lipolytic enzymes from thermophilic organisms in high-temperature environments.

Experimental analysis with *T. thermophilus* revealed the presence of two lipolytic enzymes with molecular weights of 34 kDa and 62 kDa in both intracellular and extracellular fractions, with the 34 kDa enzyme being the predominant form. The 34 kDa esterase from *T. thermophilus* HB27 exhibited optimal catalytic activity at pH 8.5 and 80 °C, showing a preference for medium-chain fatty acid esters, such as p-nitrophenyl esters with C10 chains. The enzyme displayed a half-life of 135 min at 85 °C, indicating high thermal stability. Enzymatic activity was assessed through zymography and colorimetric assays, utilizing p-nitrophenyl esters as substrates. Analyses revealed retention of 75 to 100% of activity after 30 min at 80 °C, highlighting its suitability for biotechnological applications under alkaline and high-temperature conditions.^35,36^

To date, there have been no studies focused on protein engineering aimed at enhancing the stability or activity of this enzyme. In summary, the characterization of the 34 kDa lipolytic enzyme from *T. thermophilus* HB27, with its optimal activity at elevated temperatures and alkaline pH, underscores its significant role in the enzymatic landscape of thermophilic organisms.^37^ The enzyme’s remarkable thermal stability and substrate specificity further highlight its potential biological functions. These findings set the stage for future investigations into the enzyme’s roles in thermophilic environments.

### *Bacillus subtilis* Esterase 2 (Bs2Est)

Esterases derived from *Bacillus subtilis* play a crucial role in the hydrolysis of short- and medium-chain fatty acid esters, demonstrating notable catalytic versatility.^38,39^ *Bs*2Est, an esterase from *Bacillus subtilis* DSM402, exhibits remarkable versatility in the hydrolysis of tertiary alcohols and the removal of carboxyl protecting groups, making it a valuable enzyme for industrial applications. With high thermal stability and resistance to pH variations, *Bs*2Est shows promise for biocatalytic processes, such as the enantioselective resolution of acetate esters of tertiary alcohols and the selective deprotection of peptides. This versatility underscores its significance in various biotechnological applications, including biopolymer synthesis and the development of novel methodologies in organic chemistry.^40^

Although structures for two close homologues are available, no experimentally resolved structure of *Bs*2Est has been reported to date, nor has structural information for *Bs*2Est been found in the AlphaFold database. A study investigating the enzyme’s promiscuous amidase activity used a combination of experimental assays and molecular modeling to demonstrate that amide hydrolysis is stabilized by a hydrogen-bond network involving the Glu188 residue. To evaluate this effect, mutations were introduced at this position: mutants that retained the hydrogen-bond network (E188D and E188Q) displayed a smaller reduction in amidase activity, while mutants unable to form this network (E188A and E188F) showed a more pronounced decrease in activity. These findings indicate that the hydrogen-bond network is critical for transition-state stabilization, offering a molecular basis for the enzyme’s promiscuous activity.^41^ Furthermore, the E188D variant converted the trifluoromethyl analog of 3-phenylbut-1-yn-3-yl acetate with excellent enantioselectivity (*E* > 100) yielding the (S)-alcohol with >99% enantiomeric excess.^42^ A double mutant of *Bs*2Est, E188W/M193C, demonstrated an inversion in enantioselectivity for acetylated tertiary alcohols, achieving an *E* value of 64, compared to an *E* value greater than 100 for the E188D mutant;^43^ and rapidly hydrolyze *n*-butyl, n-propyl, methoxyethyl, and allyl esters.^44^

*Bs*2Est was shown to hydrolyze incompletely converted products of the glycolysis of waste PET, producing an excess amount of TPA compared to both BHET and MHET, suggesting that the enzyme was capable of converting even PET oligomers (particularly dimers) in the incomplete glycolysis products solution to TPA, via intermediate endocleavage of dimeric PET into BHET and MHET, which were further hydrolyzed into TPA and EG. The solubility of TPA was shown to have a significant impact on the BHET-hydrolytic activity of *Bs*2Est: higher solubility of TPA in sodium phosphate buffer contributed to higher hydrolytic activity of *Bs*2Est at high concentrations of BHET compared to Tris-HCl buffer. However, in comparison, the MHET-hydrolytic activity of *Bs*2Est was considerably slower than that for BHET. *In silico* molecular docking analysis revealed that the ester group of BHET was directly oriented toward the catalytic Ser189; conversely, the carboxylic group of MHET formed a hydrogen bond with the same residue leading to an unfavorable substrate binding position.^45^

Overall, these results underscore the potential of engineering *Bs*2Est variants to enhance substrate specificity and catalytic efficiency, especially in regard to finetuning its monoesterase activity for more efficient MHET hydrolysis comparable to its BHET hydrolytic capacity.

### *Bacillus subtilis* Carboxylesterase (BsCE)

*Bs*CE is a thermophilic enzyme classified within the esterase and lipase families. This enzyme catalyzes the hydrolysis of esters with notable efficiency toward long-chain substrates and exhibits remarkable thermal stability, making it valuable for industrial processes requiring extreme conditions. *Bacillus subtilis* produces several carboxylesterases that exhibit high enantioselectivity in catalyzing the hydrolysis of esters, such has carboxylesterase NP (*Bacillus subtilis* strain Thai I-8), CesA and CesB (*B. subtilis* strain 168).^46,47^

*Bs*CE exhibits a canonical α/β-hydrolase fold, defined by a core of eight predominantly parallel β-strands enveloped by 12 α-helices, which provides structural stability essential for its enzymatic function (PDB IDs: 4CCW and 4CCY). This enzyme is organized into two principal domains: a central “core” domain that accommodates the catalytic triad (Ser130-His274-Glu245) and a “cap” domain that is integral to substrate recognition and binding. The active site is situated within a hydrophobic gorge approximately 13 Å deep, where the catalytic serine resides in a flexible loop enabling the accommodation of various substrates. Notably, structural variations in the “cap” domain significantly influence substrate specificity and enantioselectivity, allowing *Bs*CE to hydrolyze a diverse array of esters, including the enantioselective hydrolysis of naproxen methyl ester to produce S-naproxen with an enantiomeric ratio (*E*) exceeding 200.^48^

As such, the carboxylesterases from *B. subtilis* exhibit broad substrate specificity and high catalytic activity toward a diverse array of esters, including short-chain esters and triacylglycerols. *Bs*CE was purified and demonstrated maximum activity at 37 °C and pH 8.0, maintaining stability at temperatures up to 55 °C. The activities of carboxylesterases NP, CesA, and CesB were evaluated using substrates such as ibuprofen and IPG esters. While CesB (YbfK) displayed moderate enantioselectivity, its specific activity was notably lower compared to carboxylesterase NP and CesA, which are more effective in the conversion of naproxen esters. The temperature analysis revealed that both CesA and carboxylesterase NP reached peak activity at approximately 30 °C, whereas CesB achieved its maximum at 40 °C, with rapid inactivation occurring at temperatures above 55 °C. Additionally, CesB demonstrated remarkable stability at elevated pH levels, retaining 53% of its activity at pH 11, thereby underscoring its robustness under alkaline conditions.^46,47^ In a recent comparative study with three other PET hydrolases (*Hi*Cut, *Thermobifida fusca* cutinase, and a variant of *Is*PETase), *Bs*CE was shown exceptionally capable of hydrolyzing BHET with a specificity constant relatively comparable to that of natural enzyme–substrate complexes.^49^

To date, only one study has applied this rational engineering for enhancing *Bs*CE. In that study, site-directed mutagenesis was employed to produce *Bs*CE variants with enhanced thermal stability, thereby expanding its catalytic capabilities and potential applications.^50^ To enhance the thermal stability of *Bs*CE, a chimeric “cap mutant” was developed by incorporating a 30-amino-acid segment from the moderately thermostable esterase *Bs*teE. This modification, tested through CD spectroscopy and pNPA assays, yielded a modest stability increase of 4 °C and improved substrate affinity. Additional mutations (T7P, K18R, S88K, K111E) based on hydrogen bond networks further elevated stability by up to 11 °C; however, these alterations significantly reduced catalytic activity, limiting their suitability as biocatalysts.

### *Ideonella sakaiensis* MHETase (IsMHETase)

*I. sakaiensis* was first described in 2016, after being isolated from a sample of PET-contaminated sediments obtained from a PET bottle recycling unit located in the Sakai region, Japan. It is a Gram-stain-negative, aerobic, nonspore-forming rod-shaped bacterium capable of utilizing PET as its main carbon source.^51,52^ The characterization of *I. sakaiensis* revealed a distinct enzymatic machinery for the degradation and assimilation of PET composed mainly by two enzymes, responsible for the initial degradation of the polymeric substrate: *Is*PETase and *Is*MHETase. A recent proteomic characterization of *I. sakaiensis* exposed to PET revealed that, when comparing secreted enzymes, *Is*MHETase was differentially expressed and in much greater abundance than *Is*PETase, since MHET is known to inhibit the depolymerization efficiency of *Is*PETase.^53^

According to the ESTHER database, *Is*MHETase belongs to the Block X of the α/β-hydrolase superfamily, which also includes bacterial and fungal tannases and ferulic acid esterases.^23^ The 3D structure of *Is*MHETase was first described by Palm et al.^54^ through molecular replacement with a close structural homologue, a feruloyl esterase from *Aspergillus oryzae* (*Ao*FaeB). In an independent study, Sagong et al.^55^ reported the successful extracellular expression and crystallographic structure of *Is*MHETase, as well as a novel exo-PETase function. Third, Knott et al.^56^ also combined structural, computational, and biochemical approaches to describe the relationship between *Is*MHETase structure and function. Peng et al.^57^ used a computational strategy based on molecular dynamics simulations and Markov State Models to reveal interesting conformational dynamics of *Is*MHETase and describe the relationship between distal regions and active, catalytic states. Finally, Saunders et al.^58^ developed a medium-throughput colorimetric assay to screen a library of engineered variants of *Is*MHETase, aiming to increase its soluble expression and whole-cell activity through semicontinuous addition/reversion/recombination of mutations representative of different conservation thresholds from consensus sequence alignments.

*Is*MHETase is an intracellular enzyme composed of nearly 600 amino acid residues and a total molecular mass of approximately 65 kDa. The surface of the enzyme is heterogeneous and has an acidic isoelectric potential of 5.11 that well complements that of *Is*PETase (9.6).^56^ The architecture of *Is*MHETase includes a lid domain inserted between β-strand_7_ and α-helix_15_ (residues Gly251-Thr472) of the conserved α/β-hydrolase domain (Figure 3A). This lid domain partially covers the active site and also harbors a Ca^2+^-binding domain that is highly conserved in feruloyl esterases (Figure 3B) and may play a role in structural stability, although it is exceptionally large (even in comparison to its closest homologues) and contains several additional loops that are thought to be responsible for substrate specificity and affinity.^54,56^

**Figure 3:**
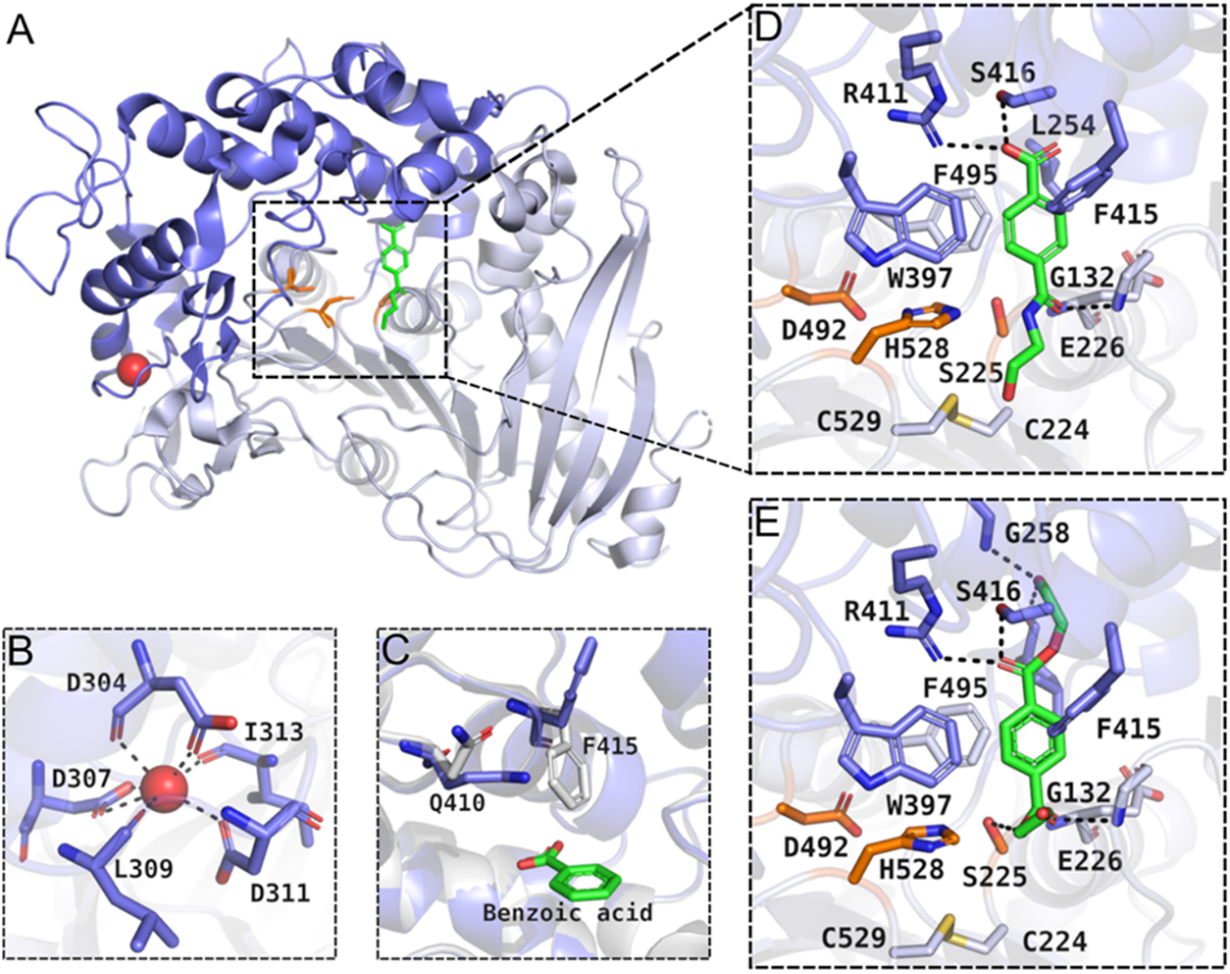
Structure of *Is*MHETase. (A) *Is*MHETase bound to MHET (green), highlighting the lid domain (blue) and catalytic triad (orange); (B) Close-up of the calcium-binding domain conserved across feruloyl esterases; (C) The concerted movement/dual occupancies of residues Gln410 and Phe415 in the apo form (blue, PDB ID 6QZ4) and ligand-bound form (white, PDB ID 6QZ3); (D) Binding mode of the nonhydrolyzable MHET analog in the active site of *Is*MHETase (PDB ID 6QGA); (E) Binding mode of BHET in the active site of *Is*MHETase (PDB ID 6JTT).

Sagong et al.^55^ point out that, while most if not all structurally similar enzymes exist as dimers, *Is*MHETase is a monomer. The authors highlight that the conformations of the lid domain involved in *Ao*FaeB dimerization are different from *Is*MHETase, which hinder the formation of a dimer but might be involved in its extracellular exo-PETase activity. The enzyme also contains five internal disulfide bonds in its structure, but the monomeric nature of *Is*MHETase may ultimately contribute to hinder its thermal stability. The enzyme has a reported *T*_m_ of 50.61 °C, but its activity rapidly decreases after 44 °C. One disulfide bridge (Cys224-Cys529) flanks the catalytic triad (formed by Ser225, His528, and Asp492) in what is called a CSHDC motif (characteristic of tannases), and the oxyanion hole comprising the backbone amide nitrogen atoms of Gly132 and Glu226.^55,59^

Much like its sister enzyme *Is*PETase, *Is*MHETase does not exhibit activity against *pNP*-aliphatic esters or aromatic esters typically catalyzed by tannase family enzymes, such as ethyl gallate or ethyl ferulate.^52^ The limited substrate preferences of these enzymes in lieu of the promiscuity in terms of the range of substrates catalyzed by other PET hydrolases and homologues, in combination with the origin from which the bacterium was first isolated and identified, support the notion that *I. sakaiensis* gradually developed its enzymes necessary for PET uptake under evolutionary constraints that shaped its activity and earned both *Is*PETase and *Is*MHETase their own E.C. numbers.^60,61^

Palm et al.^54^ elucidated the crystallographic structure of *Is*MHETase in complex with MHETA. Subsequently, Sagong et al.^55^ demonstrated that *Is*MHETase binds to BHET in an almost identical fashion, suggesting that the activity and function of this enzyme could extend beyond what was originally thought. Several mutagenesis assays have also helped to elucidate important residues involved in substrate recognition, orientation, and binding. Some of these experiments have focused on increasing the performance of *Is*MHETase, while others have aimed at tailoring the active site toward altered substrates, such as BHET and PET itself, by improving its affinity and activity for these substrates. These described mutagenesis studies and others, including their rationale and results, are summarized in Supplementary Table 1.

An induced-fit binding mechanism seems to be the consensus for *Is*MHETase. In fact, molecular dynamics simulations performed by Knott et al.^56^ revealed a concerted movement of Phe415 and Gln410 upon binding with benzoic acid (Figure 3C). In the apo form, Phe415 points away from the active site, but turns almost 180° upon ligand binding and closes the access to the active site, consolidating the interactions between protein and substrate.^54–56^ Peng et al.^57^ further proposed that the motion performed by Phe415 is in fact part of large-scale structural changes that require a longer time scale, evidenced by the conformational change undergone by surrounding loops near this residue, also transitioning between open/closed states.

Thus, MHET binds tightly to the active site of *Is*MHETase, with *K*_M_ and *k*_cat_ values of 0.0073 mM and 31 s^–1^, respectively.^52,56,62^ However, the high affinity and specificity of *Is*MHETase for benzoate structural analogues containing a negative charge in the para position in relation to the hydrolyzable ester bond can lead to product inhibition by the formation of TPA.^54^ The substrate interacts with the α/β-hydrolase domain residues Phe495, Gly132, and Ala494 via hydrophobic contacts, while being surrounded by lid domain residues Phe415, Leu254, and Trp397. Additionally, the two oxygen atoms of the free carboxyl moiety of MHET form contacts with Arg411, which is stabilized by Ser416, Ser419, and the amide group of Gly258 (Figure 3D).^54^ Similar binding patterns were observed for BHET, as mentioned earlier. The MHET moiety (which comprises the phenyl ring of the TPA moiety and the “inner” EG moiety) is stabilized by hydrophobic contacts, similar to the MHET ligand discussed (Figure 3E).^55^

While both the catalytic triad and the oxyanion hole are composed of residues of the α/β-hydrolase domain, substrate specificity seems to be almost entirely conferred by the lid domain, which is crucial for MHET hydrolysis. Knott et al.^56^ constructed a lidless *Is*MHETase by replacing the lid domain with the corresponding loop from *Is*PETase, hypothesizing that this could confer the enzyme the ability to degrade PET. However, the lidless *Is*MHETase was unable to degrade PET film, and the resulting variant had a *k*_cat_ value 1000-fold lower than the rate for the wild-type enzyme.

Conversely, Palm et al.^54^ also reported that a few mutations in the binding pocket of *Is*MHETase increased its catalytic activity toward BHET. These included dismantling the hydrogen bond network by replacing Ser416 and Ser419, as well as providing more space in the distal area of the active site by replacing Phe424. Combining these observations, Sagong et al.^55^ devised a strategy to develop an *Is*MHETase variant capable of degrading PET. For example, the addition of a R411K mutation could better stabilize the ester bond when BHET is used as a substrate instead of MHET. Finally, the authors were able to ultimately develop a *Is*MHETase variant (*Is*MHETase^R411K/S416A/F424I^) that was more active on *Is*PETase-treated PET film, confirming its exo-PETase function and BHET hydrolytic activity (*K*_M_ and *k*_cat_ values of 1.45 mM and 3.205 s^–1^ toward BHET, compared to 0.91 mM and 0.0255 s^–1^ for the WT enzyme) while still retaining some catalytic activity toward MHET.

### Mechanism

As the canonical serine hydrolase reaction mechanism of *Is*MHETase has been the most extensively studied in terms of MHET hydrolysis by these enzymes, it serves as an illustrative description in this text. The reaction mechanism of *Is*MHETase has been described using hybrid QC/MM (quantum chemistry/molecular mechanics) simulations by Knott et al.,^56^ Pinto et al.,^63^ and Feng et al.,^64^ with relatively good consensus between the authors, with differences regarding the detection of metastable, transient intermediate configurations characteristic of serine hydrolases.

The hydrolytic reaction mediated by *Is*MHETase thus operates according to the typical mechanism of serine hydrolases,^65^ with two major steps that involve the formation of an acyl-enzyme intermediate (acylation) followed by the hydrolytic release of products in the following stage (deacylation) (Figure 4). The acylation step involves several consecutive reactions. First, a proton is transferred from the catalytic Ser225 to His528, followed by a nucleophilic attack and the formation of an O–C bond between Ser225 and the carbonyl of MHET. This is followed by the cleavage of the MHET ester bond, facilitated by the formation of a short-lived tetrahedral intermediate. Free energy calculations have estimated free energy barriers for the acylation step of 13.09 ± 0.17,^56^ 9.42 ± 0.13,^63^ and 10.7 ± 3.4 kcal/mol.^64^

**Figure 4:**
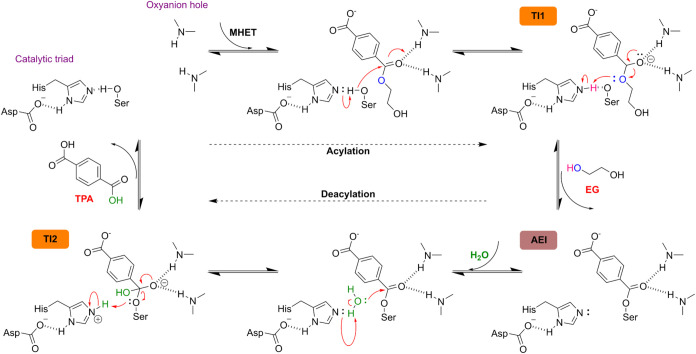
Hydrolytic mechanism of *Is*MHETase, based on the canonical reaction mechanism of serine hydrolases.^65^ Upon MHET binding, histidine prompts the deprotonation of the catalytic serine, activating it for the nucleophilic attack on the carbonyl atom of MHET, resulting in the first tetrahedral intermediate (TI1) and the formation of an acyl-enzyme intermediate (AEI). As EG leaves the active site, a water molecule is deprotonated by histidine and a second nucleophilic attack occurs, prompting the formation of a second tetrahedral intermediate (TI2). Its collapse regenerates the active site for a new reaction cycle. Adapted with permission from ref 65. Copyright 2015 American Chemical Society.

The collapse of the first tetrahedral intermediate then leads to the release of EG, which had remained in stable interaction with His528, and the formation of an acyl-enzyme intermediate.^56,63^

The release of EG, which has a high propensity to naturally and quickly diffuse to the solvent as shown by short classical molecular dynamics simulations,^56,63^ allows access of bulk waters to the active site and subsequent deprotonation of a nearby water molecule by His528, followed by the nucleophilic attack of the water hydroxyl group to the carbonyl group of MHET, resulting in the formation of a second tetrahedral intermediate. This nucleophilic attack and the collapse of this second tetrahedral intermediate then yields TPA. The proton is transferred from His528 to Ser225, regenerating the active site for the next catalytic cycle. In all three studies, this deacylation step was found to be rate-limiting, with free energy barriers of 19.8 ± 0.10,^56^ 19.35 ± 0.15,^63^ and 25.3 ± 3.40 kcal/mol.^64^

### Mle046

Mle046 is a MHETase-like enzyme from an uncultured bacterium identified in metagenomic data from a poly­(butylene adipate-*co*-terephthalate) (PBAT)-enriched marine microbial consortium, most likely belonging to an Alphaproteobacterial plasmid. Mle046 was found to be upregulated in the metatranscriptome of their sample through most of the time series, highly expressed in both film-attached and free-living microbial communities. The authors describe its putative role in the degradation of a PBAT-based film, where periplasmic hydrolases break down the film into oligomers and monoesters of mono­(hydroxybutyl) terephthalate, which is broken down by Mle046 into terephthalic acid and 1,4-butanediol, akin to the biological degradation of PET plastic.^66^

Mle046 is a mesophilic enzyme sharing approximately 47% identity with *Is*MHETase, with a theoretical molecular mass of approximately 60 kDa. The enzyme rapidly degrades MHET to TPA and EG at temperatures varying between 20 and 40 °C, and still retains a considerable amount of activity at a wide range of temperatures: the enzyme exhibits approximately 50% activity at 5 °C (making it a cold-active enzyme) and still retains some activity even at 60 °C, substrate concentrations, and pH conditions. Mle046 kinetics were shown to follow a Michaelis–Menten curve with concentrations up to 2.23 mM, with *K*_M_ and *k*_cat_ values estimated to be 2.63 ± 0.797 mM and 80.9 ± 15.8 s^–1^, respectively, at 30 °C. Although its affinity for MHET was lower than that of *Is*MHETase, the turnover rate of MHET by Mle046 was considerably higher; due to this discrepancy, the catalytic efficiency of Mle046 is indeed lower than that of WT *Is*MHETase, but in similar order of magnitude to homologous MHET hydrolytic enzymes.^67^

### *Candidatus* Bathyarcheota PET46

PET46 is an archaeal promiscuous feruloyl esterase encoded in a marine Bathyarchaeota metagenome-assembled genome. This monomeric enzyme also shares the canonical α/β-hydrolase fold: eight β-strands connected by seven α-helices comprise the core of the enzyme, while three α-helices and two antiparallel β-strands make up the 45 amino acid residues-long lid domain. Despite low sequence identity, the structure of PET46 superimposes relatively well with *Is*PETase and LCC, with the largest differences being the presence of a lid domain in the former, as well as extended and truncated loops between β_4_ and α_3_ and β_10_ and α_10_ (which carries the His residue of the catalytic triad), respectively. The enzyme also lacks a residue equivalent to Trp185 in *Is*PETase or Trp190 in LCC, suggesting that the lid domain of PET46 is responsible for the aromatic clamping needed for effective substrate binding and stabilization. As such, Perez-Garcia et al.^68^ constructed a lidless variant of PET46 replacing it for the Trp185-carrying loop from *Is*PETase and incubated both the WT, the lidless variant, and three single-point mutants (A46V, A140I, and K147A) at 30, 60, and 70 °C to assess its PET hydrolytic activity.

WT PET46, as well as the K147A and A46V variants, degraded all PET trimers to MHET and TPA within the first 3 h of reaction, with the latter producing 3.2 times more products at 30 °C than the WT enzyme during the first 24 h. As PET46 is classified as a feruloyl esterase and it was shown to degrade both BHET and MHET, the authors propose that the enzyme degrades PET through an exo-PETase mechanism, where hydrolysis happens at the extremity of the polymer chain, where the PET trimer is cleaved to MHET units that are subsequently converted to TPA and EG. The enzyme was inactive on PET foil, but incubation of 3 μM enzyme with semicrystalline PET powder released 1624.14 μM aromatic products after 3 days at 60 °C, 99% of which was TPA (corresponding to 3.38% conversion). The PET hydrolytic activity of PET46 was comparable to that of LCC, but its BHETase and MHETase functions were remarkably superior. At 70 °C, PET46 was 40.9 and 215.7% more active on BHET than *Is*PETase at 30 °C and LCC at 50 °C, respectively. With MHET as the substrate, PET46 was up to 27.7 and 86.3% more active than the reference enzymes.^68^

### *Geobacillus stearothermophilus* KL-MHETase (*G. stearothermophilus* Est30)

KL-MHETase is a computationally designed MHET hydrolase, based on a search in the PDB structural database for thermophilic scaffolds with promiscuous activity toward MHET hydrolysis, followed by the computational redesign of their active site. Zhang et al.^69^ experimentally confirmed the activity of five thermophilic scaffolds at 50 °C, and selected a carboxylesterase (Est30) from *G. stearothermophilus* for in silico redesign as the WT enzyme showed 1.33 times higher MHET hydrolytic activity than FAST-PETase, and its PET degradation activity reached 1/7 that of FAST-PETase, indicative of its high specificity toward MHET.

Est30 is a thermophilic carboxylesterase with optimal hydrolysis of short acyl ester chains at 70 °C. The enzyme, comprising approximately 247 amino acid residues, folds into two domains—the larger core α/β-hydrolase domain comprising seven mostly parallel central β-strands surrounded by six α-helices, and a smaller lid domain consisting of three α-helices. The binding domain is located in the interface between both domains, containing the catalytic triad formed by Ser94-His223-Asp193 and the oxyanion holes comprising Phe25 and Leu95. The binding site extends on both sides of the catalytic serine in a groove located in the interface between the lid and hydrolase domains and befits a polar ligand.^70^

To obtain more active variants of Est30, the active site of the enzyme was redesigned to stabilize the catalytically productive transition state conformation of MHET to tightly bind the carboxyl terminus of the tetrahedral intermediate through the construction of a hydrogen bonding network. Mutants were screened, optimized and selected through high-throughput molecular dynamics simulations, and a total of 14 variants were selected for experimental validation of their MHET hydrolytic activities at 50 °C. KL-MHETase (also labeled M8, Est30^I171K/G130L^) achieved 36-fold higher catalytic activity than WT Est30, mainly due to an enhanced catalytic rate (from *k*_cat_ = 1.17 s^–1^ to *k*_cat_ = 21.43 s^–1^), as well as its stability at 50 °C, retaining 75% of its initial activity after 96 h of incubation, making it most compatible with engineered PET hydrolytic enzymes such as FAST-PETase.^69,71^

The most active variant, M14, included the additional mutation M127S, but its melting temperature was considerably lower (63.29 °C) than M8 (67.58 °C). All three mutations are located in the lid domain of Est30; I117 K and M127S provide additional hydrogen bonding partners to the carboxyl moiety of MHET but potentially disrupt the hydrophobic surrounding environment and thus slightly hinder its thermostability, while G130L enhances hydrophobic stacking effect with the benzene ring of MHET to stabilize the substrate in catalytic geometry.

### *Maribacter* sp. Carboxylesterase (MarCE)

*Mar*CE is carboxylic acid hydrolase with a molecular mass of 58 kDa identified in a *Maribacter* sp. strain previously isolated from the marine sponge *Stelligera stuposa*, shown to possess hydrolytic activities against polyesters such as tributyrin, polycaprolactone diol, and polycaprolactone.^72,73^ MarCE shares approximately 72.90 and 70.76% similarity to *Bs*EstB and *Tf*Ca, respectively, although its mean identity with the latter is relatively low (27.08%).

Considering the similarity between these enzymes, Carr et al.^72^ performed the molecular docking of BHET into the active site of an AlphaFold model of *Mar*CE, followed by superimposition of the predicted structure with a crystallographic structure of *Tf*Ca. *Mar*CE is structurally similar to other α/β-hydrolases, featuring 12 mixed central β-sheets, although its lid domains are smaller. This mesophilic enzyme was shown to be active on BHET via incubation with 2 mM of the substrate at 30 °C for 2 h, yielding 1.28 mM MHET and 0.12 mM TPA.^72^

### *Bacillus subtilis* PET-86 (BsEst) and *Chryseobacterium* sp. PET-29 (ChryBHETase)

Both enzymes were identified from the environment through enzymatic mining from the collection and processing of 50 samples from reuse landfills, including postconsumer PET waste and surrounding soil. In their comprehensive study, Li et al.^74^ used diethyl terephthalate as a model substrate to screen for potential PET-degrading microorganisms. *In vitro* characterization of these two enzymes with PET powder, BHET, and p-nitrophenyl esters further revealed their preference for BHET as a substrate compared to aliphatic esters. Furthermore, the *Bs*Est enzyme identified in this study shares 97.5% identity with *Bs*2Est and 37.21% with *Tf*Ca, while *Chry*BHETase shares 25.55, 24.66, 12.41, and 14.31% with *Bs*2Est, *Tf*Ca, *Is*PETase, and *Is*MHETase, respectively.

The authors performed molecular dynamics simulations of both enzymes in the absence and of BHET (dispersed within the solvation box) and observed that water molecules tended to accumulate within the substrate binding cleft between the lid domain and the α/β-hydrolase domain of both enzymes, which could possibly block the larger BHET molecule. Based on this, Li et al.^74^ developed variants of *Bs*Est and *Chry*BHETase by truncating “barrier regions” and replacing them with flexible glycine–glycine linkers, and both Δ*Bs*Est and Δ*Chry*BHETase variants exhibited improved catalytic activity than their corresponding wild-types: Δ*Bs*Est had a 2-fold improvement in BHET conversion compared to WT *Bs*Est, while Δ*Chry*BHETase achieved over 90% TPA yield from BHET within 12 h, translating into 3.5-fold and 1.5-fold enhancements in catalytic efficiency, respectively, and meaning that these enzymes likely cleave BHET into MHET and MHET into TPA.^74^

## Improving Pet Degradation through Enzymatic Cocktails

In recent studies, the synergistic cooperation of functionally complementary enzymes in the degradation of PET has been extensively showcased. PET hydrolases generally exhibit significantly hydrophobic active sites, highly receptive to polymeric or oligomeric molecules.^77,78^ As a result, comparatively smaller intermediates still bind tightly to these enzymes, but the noncatalytic binding pose either slows down hydrolysis or effectively functionally inhibits the enzymatic reaction, leading to reduced catalytic turnover.^69^ This nonproductive inhibition, distinct from classical allosteric effects, stems from the inability of a given enzyme to efficiently convert intermediate products (particularly MHET), resulting in accumulation of intermediate products and decreasing polymer-to-monomer conversion yields.

For instance, although thermophilic cutinases are among the most efficient PET-degrading enzymes, even the benchmark LCC (and its engineered variant LCC^ICCG^) do not hydrolyze MHET at a fast enough rate, leading to its accumulation in the reaction system and diminished overall depolymerization rates.^79–81^ Arnal et al.^82^ compared the catalytic hydrolysis of four engineered PET hydrolases with an industrial scale-up in mind. While LCC^ICCG^ outperformed HotPETase,^83^ FAST-PETase,^71^ and PES-H1^L92F/Q94Y^,^84^ depolymerizing 97% of 16.5% (w/w) postconsumer waste PET into TPA and EG in 24 h at 68 °C, the authors note that 79% depolymerization was achieved in just 6 h, highlighting that most of the remaining time was devoted to the slower hydrolysis of MHET. Barth et al.^85^ also found binding constants of 0.568 L·mmol^–1^ for MHET and 0.550 L·mmol^–1^ for BHET as “competitive inhibitors” of *Tf*Cut2, but a significantly lower degradability of MHET. Thus, by incorporating an enzyme that can remove the intermediate product MHET from the system, it is possible to alleviate the observed functional inhibition of PET conversion systems mainly caused by the accumulation of intermediate products.

Although hydrolysis of BHET by *Tf*Ca is 500 times faster than that of MHET,^28^ immobilized *Tf*Ca has been used in conjunction with free *Thermobifida fusca* KW3 cutinase (*Tf*Cut2) and LCC at 60 °C over a reaction time of 24 h.^86^ While 60 °C was shown to be the optimal temperature for the hydrolysis of PET trimers, the enzyme rapidly loses activity in these conditions.^27^ Immobilization of *Tf*Ca on SulfoLink resin, therefore, should supposedly increase its stability in dual enzyme systems for a longer period of time. The inclusion of immobilized *Tf*Ca in the reaction system resulted in a 2.4-fold higher release of products from the degradation of amorphous PET film in combination with LCC than with *Tf*Cut2, or a 91 and 104% increase in the total amounts of products (as the sum of BHET, MHET, and TPA) released in combination with *Tf*Cut2 and LCC, respectively. Amounts of MHET in the reaction system were observed to decrease proportionally to the amount of immobilized *Tf*Ca, while BHET contributed only 1% of the total amount of hydrolysis products.^86^ von Haugwitz et al.^28^ also incubated both WT *Tf*Ca and the engineered variant *Tf*Ca WA with a thermostable penta mutant of *Is*PETase^87^ in dual enzyme systems to degrade PET nanoparticles, crystalline PET powder, and amorphous PET film at 45 °C. When only the *Is*PETase variant was used, MHET was the main product independently from the initial amount of PET nanoparticles, but the inclusion of WT *Tf*Ca or *Tf*Ca WA enabled the full conversion of MHET into TPA. For each type of PET-based substrate (nanoparticles, powder, and film), final TPA yield increased by 8.3, 14, and 11-fold when *Tf*Ca was used in combination with the *Is*PETase penta mutant.^28^

To evaluate the efficiency of the synergistic cooperation of both *I. sakaiensis* enzymes, Knott et al.^56^ conducted a study comparing the overall activity of free *Is*PETase, *Is*MHETase, and chimeric *Is*PETase:*Is*MHETase proteins in two-enzyme systems. While overall degradation rates scaled with *Is*PETase loading, the mere inclusion of *Is*MHETase in the reaction system results in a significant improvement of depolymerization, even without increasing the *Is*PETase loading. The degree of improvement in depolymerization was found to be proportional to the amount of *Is*MHETase added to the reaction system. Furthermore, *Is*PETase:*Is*MHETase chimeric proteins, connected by glycine-serine peptide linkers of varying lengths, also exhibited even greater performance than the free enzymes in mixed reaction systems, outperforming *Is*PETase, and demonstrating higher catalytic activity toward MHET, likely due to the greater availability of nearby catalytic sites arising from the proximity between the MHET released in the first step and the linked *Is*MHETase, and the promotion of substrate channeling.

As a semicrystalline thermoplastic polymer, PET contains both crystalline and amorphous regions, characterized by highly ordered, uniformly packed molecules and randomly arranged molecular chains, respectively. Previous research has indicated that enzymatic hydrolysis of the amorphous regions of PET is highly favored by PET hydrolases due to amplified chain mobility.^88^ The application of thermophilic and engineered thermostable enzymes over mesophilic counterparts is also seen as favored due to the enhanced mobility of PET polymer chains at the glass transition temperature (*T*_g_), resulting in higher degradation rates.^89^ To enhance the performance of the PET degradation process, Chen et al.^90^ utilized the SpyTag/SpyCatcher system^91^ to coimmobilize a thermostable variant of *Is*PETase, named DuraPETase, and WT *Is*MHETase in calcium phosphate nanocrystals. The DuraPETase variant has a reported melting temperature 31 °C higher than WT *Is*PETase and exhibits a 300-fold increase in semicrystalline PET degradation at mild temperatures.^92^ The authors reported similar results to those observed by Knott et al.:^56^ the coimmobilized enzymes showed higher degradation efficiency than the free two-enzyme system, and immobilization also helped to maintain the stability and activity of the enzymes for a longer period of time. The PET degradation efficiencies of the coimmobilized system were 9.7 and 5.2-fold higher than those of the free enzyme system at 40 and 50 °C after 6 days, respectively. At 40 °C, all the MHET released by DuraPETase was hydrolyzed into TPA. At 50 °C and higher, however, a significant accumulation of MHET was reported due to both WT *Is*MHETase inability to match the degradation efficiency of DuraPETase at higher temperatures and its poor tolerance to elevated temperatures, highlighting the need for further studies into the development of thermostable *Is*MHETase variants for practical biotechnological applications.

Aside from temperature conditions in which enzymatic degradation reactions are carried out, the pretreatment of PET via amorphization to reduce the degree of crystallinity of the polymer is also believed to be a way to improve the efficiency of said process. A study conducted by Tarazona et al.^93^ offers an approach to evaluate how mechanisms such as surface amorphization, the plasticizing effects of water on the polymeric substrate, and the difference between the *T*_g_ on the surface layer in comparison to the *T*_g_ in the polymer bulk can affect the biocatalysis process mediated by PET hydrolases. The authors also studied the synergism between a *Is*PETase triple mutant^87^ and *Is*MHETase^W397A^. Among their findings, the authors describe that the addition of *Is*MHETase^W397A^ increases the degradation of thin PET films by approximately 20%, and the reaction proceeds five times faster at 50 °C in comparison to the two-enzyme system at 40 °C. However, the enzymes denatured rapidly at 60 °C.

The authors also reinforce that two of the main factors affecting the depolymerization rates of PET polymer are the deposition of deactivated enzymes on its surface blocking further degradation of the PET film and product inhibition. The addition of MHET hydrolytic enzymes mitigates the latter for the most part, but the development of systems focused on continuous removal of released products and inactivated enzymes—or extending their activity, such as through immobilization—seems to be an interesting avenue.

Han et al.^94^ constructed an engineered bacteria using cell-surface display of several PET hydrolases using different anchor proteins for the hydrolytic treatment of PET plastics. Cell-surface display of proteins involves the fusion of the terminus of a protein of interest with an anchoring protein,^95^ so that the target protein will be expressed on the cell membrane. The codisplayed *Is*MHETase and recently developed FAST-PETase variant^71^ system, using PgsA anchor protein on the surface of *Escherichia coli*, exhibited the highest PET hydrolysis rate. Notably, the order of the fused proteins C-terminus end also had influence on the makeup of hydrolysis products, with either FAST-PETase or *Is*MHETase located on the C-terminus leading to higher PET hydrolysis and MHET hydrolysis, respectively, likely due to enzyme contact with the substrate. Nevertheless, corroborating previous findings, the authors point out that regardless of which enzyme was located on the C-terminal, the combined FAST-PETase:*Is*MHETase system showed higher hydrolytic capabilities than the PET hydrolase alone due to the alleviation of the decline in hydrolysis caused by the release and accumulation of the reaction product.

Zhang et al.^69^ also combined FAST-PETase^71^ with the computationally designed KL-MHETase for the depolymerization of postconsumer bottle grade PET powder over 24 h of reaction at 50 °C at various enzyme concentrations. The authors observed that the sum of products from the conversion of PET scaled with FAST-PETase loading, but the concentration of TPA in total products depended on KL-MHETase loading; however, efficient conversion of PET decreased with increased KL-MHETase loading due to the latter blocking the substrate-binding cleft of FAST-PETase. Nevertheless, maximum TPA yield was found on a 2:6 ratio between KL-MHETase and FAST-PETase loading. Similarly to the dual-enzyme system constructed by Knott et al.,^56^ the authors engineered fusion enzymes connected by flexible glycine and serine linkers of varying lengths (20, 28, and 36 residues) as well a rigid linker (4 residues) to covalently link the C-terminal of KL-MHETase to the N-terminal of FAST-PETase. The PET degradation activity of these fusion enzymes was significantly dependent on the linking order between enzymes, the specific amino acid sequence of the linkers, and their lengths. The composition of TPA in the final products increased to 99.5% with a 36 residues-long linker (KL36F), 1.47-fold higher than when FAST-PETase was used alone. Increasing the size of PET powder also increased its conversion rate, and the catalytic rate of the fusion enzyme KL36F was 52.6-fold higher than that of FAST-PETase with regard to MHET hydrolysis. The authors also compared the performance of FAST-PETase and the fusion enzyme in higher concentrations and volume of PET. The purity of TPA again exceeded 99%, while MHET concentration in the single enzyme system reached up to 20 mM (5 times higher), leading to accumulation and stagnant conversion of PET due to product inhibition. In summary, these studies show that controlling the complex structure of dual enzyme systems through the introduction of a peptide linker between enzymes can alleviate effects from steric hindrance and protein–protein interactions.

Li et al.^74^ combined engineered variants Δ*Bs*Est and Δ*Chry*BHETase with several thermophilic PET hydrolases, including DepoPETase, FAST-PETase, DuraPETase, ThermoPETase, LCC, and LCC^ICCG^, as well as the mesophilic WT *Is*PETase. In all cases, dual enzyme systems resulted in substantially higher release of TPA compared to PET hydrolases alone, where significant accumulation of BHET and MHET could be detected even after 96 h at 60 °C. Specifically, as the authors point out, the DepoPETase-Δ*Bs*Est, FAST-PETase-Δ*Bs*Est, and FAST-PETase-Δ*Chry*BHETase combinations yielded 1.8, 2.0, and 1.6-fold more TPA than any of the *Is*PETase variants in single-enzyme systems.

Lu et al.^96^ developed a sequential reaction system using a previously engineered variant of *Thermobifida alba* AHK119 cutinase^19^ (muEst1) in combination with KL-MHETase. First, PET was fully depolymerized by muEst1 at 65 °C for 48 h, followed by 12 h of KL-MHETase-mediated degradation of released intermediate products at a temperature of 50 °C, so that both enzymes could operate at their optimal temperatures. Therefore, multistage cascade reactions could also be considered to overcome the challenges of different reactions conditions of PET and BHET/MHET hydrolases to the in tandem use of synergistic enzymes.

## Microbial Metabolism of Pet Degradation Products

To develop biotechnological methods for the upcycling of PET and other materials, it is essential to understand the pathways through which bacteria can convert complex, recalcitrant substrates into metabolites. This involves elucidating the structures and characterizing the biochemical mechanisms and pathways involved in the process.^97^ As discussed throughout this review, the depolymerization of PET typically yields soluble intermediates. These lower molecular weight products can cross the bacterial cell wall and diffuse into the periplasm or cytoplasm, where specific hydrolases can further catalyze their conversion into the fundamental PET monomers: TPA and EG.^52^

Although TPA is similar to plant-derived aromatic compounds, it is not a very common substrate used for bacterial growth. *I. sakaiensis* was found to harbor a gene cluster significantly similar to two gene clusters identified in *Comamonas sp.* E6,^52^ which has served as an archetypal degrader of plastic-derived monomers, and has been shown to be capable of utilizing phthalate isomers as sole carbon and energy sources.^98^ This gene cluster in *I. sakaiensis* corresponds to two sets of protein homologues in *Comamonas sp.* E6 that contribute to the TPA to protocatechuic acid (PCA) conversion pathway, namely the tphR transcription factor, the tphC TPA periplasmic binding receptor, the tphA1 reductase and tphA2A3 oxygenase components of terephthalic acid dioxygenase (TPADO), and the 1,2-dihydroxy-3,5-cyclohexadiene-1,4-dicarboxylate (DCD) dehydrogenase.^99^ As shown in Scheme 1, TPA resulting from the cleavage of MHET is transported to the cytoplasm where TPADO, together with its cognate reductase, dihydroxylate and dearomatize TPA into DCD. Zinc-dependent DCD dehydrogenase then decarboxylates the substrate to ultimately yield PCA.^97^

**Scheme 1:**
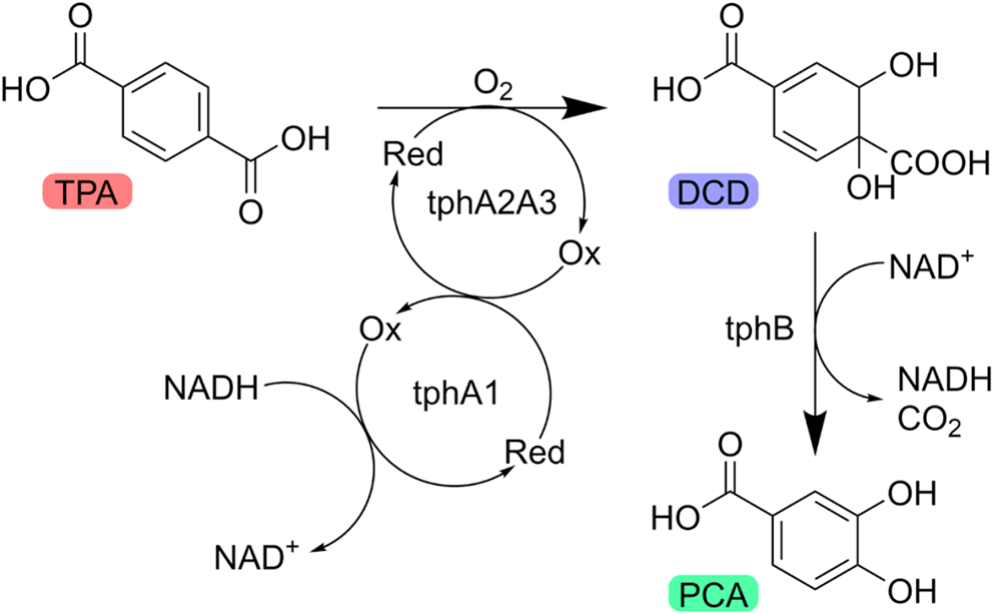
Conversion of TPA to PCA

There are three characterized pathways for the further degradation of PCA depending on the location of the initial ring-opening oxidation before it can be assimilated into the tricarboxylic acid cycle by organisms catabolizing aromatic compounds, namely the 3,4-*ortho*, 2,3-*meta*, and 4,5-*meta* cleavage pathways (Scheme 2).^100^ Yoshida et al.^52^ further describe the presence of a set of genes whose products share 52 and 60% identity with the α- and β-subunits of PCA 3,4-dioxygenase (pcaHG) from *Pseudomonas putida*, suggesting that the metabolism of PCA by *I. sakaiensis* might follow the same route, with pcaHG starting the conversion of PCA to 3-carboxy-cis,cis-muconate (β-carboxymuconate) which converges to β-ketoadipate and ultimately yields succinate and acetyl-CoA.^101^

**Scheme 2:**
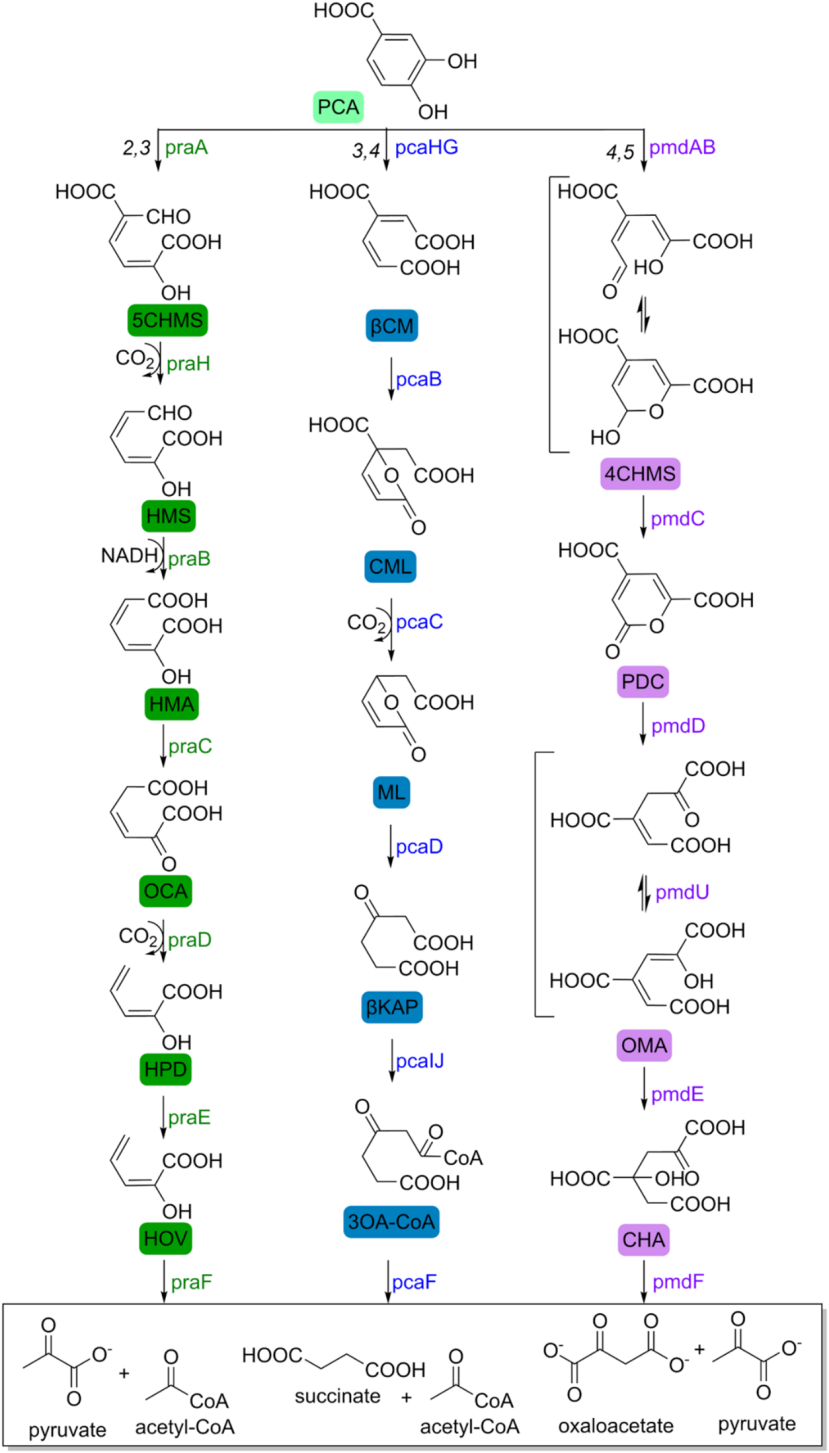
PCA Metabolism through the 2,3-*meta* (Green), 3,4-*ortho* (Blue), and 4,5-*meta* Cleavage Pathways^a^

a 5CHMS, 5-carboxy-2-hydroxymuconate-6-semialdehyde; HMS, 2-hydroxymuconate-6-semialdehyde; HMA, 2-hydroxymuconate; OCA, 4-oxolacrotonate; HPD, 2-hydroxypenta-2,4-dienoate; HOV, 4-hydroxy-2-oxovalerate; βCM, β-carboxymuconate; CML, carboxymuconolactone; ML, muconolactone; βKAP, β-ketoadipic acid; 3OA-CoA, 3-oxoadipyl-CoA; 4CHMS, 4-carboxy-2-hydroxymuconate-6-semialdehyde; PDC, 2-pyrone-4,6-dicarboxylate; OMA, 4-oxalomesaconate (keto and enol forms); CHA, 4-carboxy-4-hydroxy-2-oxoadipate.

Conversely, the para-cleavage pathway of PCA initiated by 2,3-dioxygenase (PraA) begins with the conversion of PCA into 5-carboxy-2-hydroxymuconate-6-semialdehyde (5CHMS), followed by decarboxylation and dehydrogenation by 5CHMS decarboxylase (PraH) and HMS dehydrogenase (PraB), to yield pyruvate and acetyl-CoA.^102^ Similarly, in the 4,5-cleavage pathway, PCA is first converted to 4-carboxy-2-hydroxymuconate-6-semialdehyde (4CHMS) by 4,5-dioxygenase (PmdAB), and is then spontaneously converted to an intramolecular hemiacetal form before undergoing oxidation by 4CHMS dehydrogenase to produce 2-pyrone-4,6-dicarboxylate (PDC), and ultimately yields pyruvate and oxaloacetate.^103,104^

EG is a natural product that can be metabolized by many microorganisms via two distinct pathways. In acetogens and in a few anaerobic organisms, EG is first dehydrated to acetaldehyde likely by the PduC subunit of 1,2-propanediol dehydrogenase, which is either reduced using nicotinamide adenine dinucleotide (NADH) or oxidized in a CoA-dependent reaction by propionaldehyde dehydrogenase (PduP), thereby transferring electrons onto NAD^+^, and disproportionating aldehyde into ethanol and acetyl-CoA.^105^ Ethanol can then be oxidated to acetate in a second step, and this pathway is linked to adenosine triphosphate (ATP) and NADH production (Scheme 3A).^106^ In contrast, in the pathway of bacteria such as *P. putida* and *I. sakaiensis*, EG is oxidized into glycolaldehyde by a set of periplasmic pyrroloquinoline quinone alcohol dehydrogenases (PedE and PedH, as well as XoxF in *I. sakaiensis*), which is further oxidized by an aldehyde dehydrogenase (PedI) into glycolate. The latter is then converted into glyoxylate by a glycolate oxidase (GlcDEF).^107^ Finally, glyoxylate can be converted into glycerate and then pyruvate (Scheme 3B), which is then converted into acetyl-CoA by enzymes of the pyruvate dehydrogenase complex before entering the tricarboxylic acid cycle.^108,109^

**Scheme 3:**
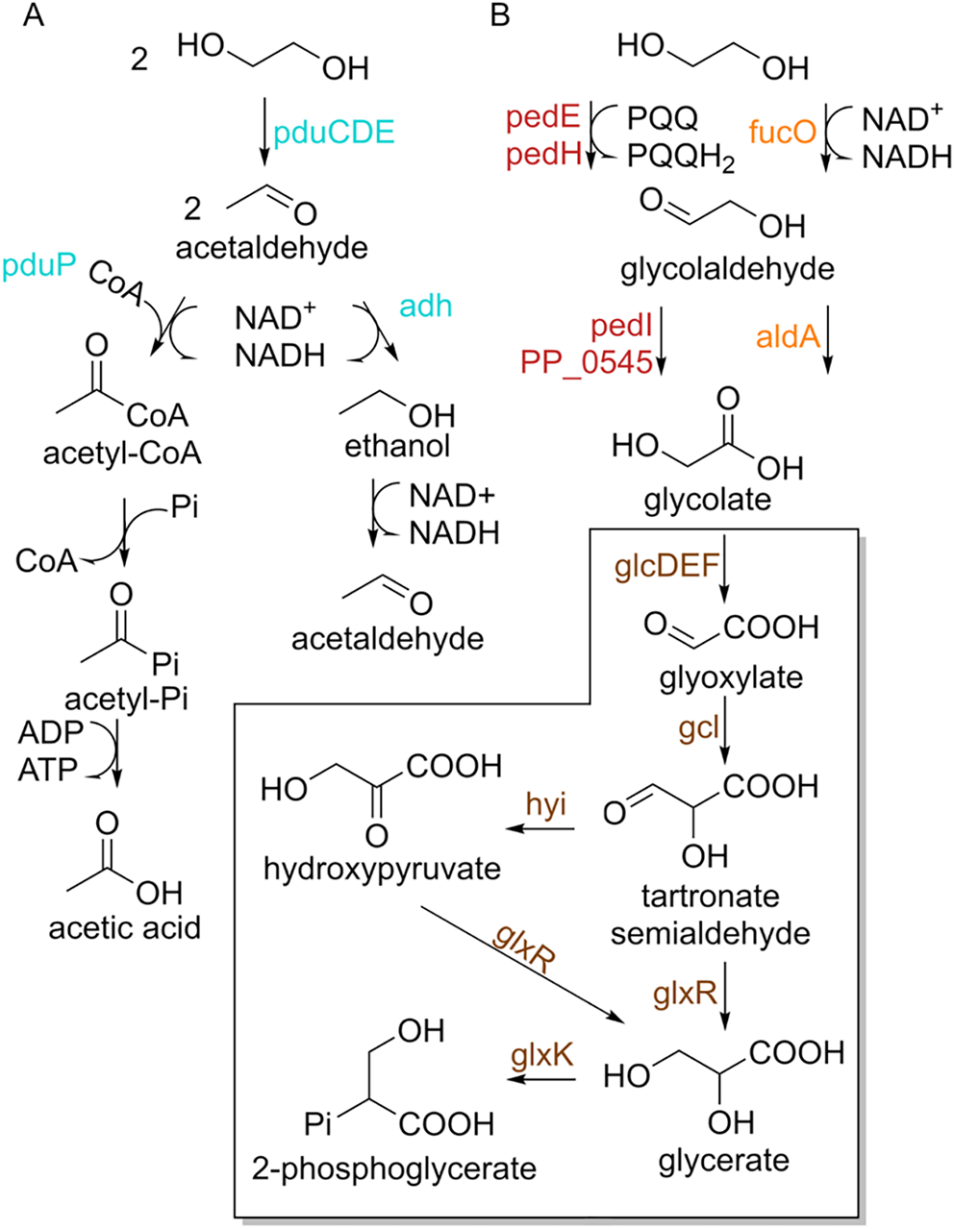
EG Metabolism in Microbes^a^

a (A) Anaerobic catabolism of EG in the acetogenic bacterium *Acetobacterium woodi*; (B) Sequential oxidation of EG to glycolate. For reference, the metabolic pathways in *P. putida* and *E. coli* are depicted in red and orange, respectively. Genes in the glycerate pathway are colored brown.

## Approaches for the Valorization and Upcycling of Pet Waste

In different industries, genes of heterologous enzymes are being introduced into microbial hosts with increased frequency and, depending on the complexity of the metabolic pathway or the target product, they can enable the up-cycling and production of valued-added substances from cheap or recycled feedstock.^110^ Loll-Krippleber et al.^111^ developed a whole-cell biocatalyst using the yeast *Saccharomyces cerevisiae* as a chassis for the expression of *Is*MHETase and the conversion of MHET into TPA and EG, without the need to purify the expressed MHET hydrolytic enzyme. Further direct biodegradation of PET by artificial microbial consortia has also been explored using synthetic biology. A four-species microbial consortium was constructed with two engineered *B. subtilis* strains that could secrete heterologous PETase and MHETase from *I. sakaiensis*, accompanied by TPA and EG-uptaking *Rhondococcus jostii* RHA1 and *P. putida* KT2440, respectively. The use of microbial consortia can reduce the metabolic burden compared to the utilization of a single microorganism and alleviate effects resulting from product inhibition, as shown by a 17.6% improvement in PET film weight loss compared to the two engineered *B. subtilis* species alone within 3 days.^112^

Moreover, Moog et al.^113^ successfully expressed *Is*PETase using the marine microalga *Phaeodactylum tricornutum* and found that the secreted engineered *Is*PETase was able to maintain its PET depolymerizing activity in a saltwater-based environment even at low mesophilic temperatures, which could serve as a proof of concept and potentially pave the way for bioremediation of microplastics present in polluted waters. The recent discovery of marine PET, BHET, and MHET hydrolases could also reinforce this idea given the complex marine environment.^66,68,72,113^

Strains of *P. putida* (GO16, GO19) and *P. frederikbergensis* GO23 have been studied for their ability to metabolize EG and TPA derivatives. Their role in the upcycling of PET into the biodegradable polymer polyhydroxyalkanoate (PHA) has been demonstrated before.^114^ In that study, waste PET was pyrolyzed to produce TPA (while the remaining fractions were incinerated for energy recovery), which served as feedstock for bacterial growth and production of PHA. This polymer is a range of diverse polyesters of (*R*)-3-hydroxyalkanoic acids, typically produced by specific Gram-positive or Gram-negative bacteria in the form of cytoplasmic inclusions as a way to store carbon under specific growth conditions or as a response to a range of environmental stresses.^115^ The intrinsic properties displayed by PHA-based plastic films have attracted interest to a range of applications, including food packaging.^116^

Fujiwara et al.^117^ reported the identification of a gene cluster in *I. sakaiensis* encoding enzymes highly similar to those produced by *Cupriavidus necator* H16, known for producing and accumulating great quantities of poly­(3-hydroxybutyrate) (PHB).^118^ The gene cluster comprising phaC, phaA, and phaB (encoding PHA synthase, β-ketothiolase, and acetoacetyl-CoA reductase, respectively) is crucial for converting acetyl-CoA into PHAs. After optimization of cultivation conditions of *I. sakaiensis*, the bacteria was shown to accumulate PHA from PET up to 48 ± 5% of dry cell weight, equivalent to a PHA titer of 0.75 ± 0.09 g/L, mostly in the form of methyl 3-hydroxybutyrate and trace amounts of 3-hydroxyvalerate.^117^

Similarly, Werner et al.^119^ used a set of sequential genetic engineering efforts in *P. putida* KT2440 to design a whole-cell biocatalyst to enable the conversion of PET hydrolysis products into β-ketoadipate through the incorporation of a TPA catabolism pathway sourced from *Comamonas sp.* E6 and a transporter from *R. jostii*. The authors depolymerized PET into BHET via chemocatalytic glycolysis using EG and titanium butoxide and constructed a *P. putida* strain expressing both *Is*PETase and *Is*MHETase (strain RC038) to further enzymatically convert BHET into TPA and EG. As mentioned earlier, *P. putida* strains metabolize PCA through the 3,4-cleavage pathway. By deleting the *pca*IJ genes (generating strain AW165) that encode the two subunits of 3-oxoadipate-CoA-transferase, the authors enabled the accumulation of β-ketoadipate, which can be reacted with hexamethyl diamide to produce a polyamide analogous to nylon-6,6 with higher molecular weight, glass transition temperature, and melting temperature than nylon-6,6 produced from adipic acid under similar conditions.^119,120^

However, medium supplementation with glucose was necessary to support cell growth. The previous engineered *P. putida* strain was further optimized via the combined heterologous expression of the *dcaAKIJP* operon (encoding initial uptake and activation steps for dicarboxylates) from *Acinetobacter baylyi* and deletion of genes encoding native repressors of phenylacetate catabolism (PaaX) and β-oxidation reactions (PsrA).^121^ The resulting *P. putida* strain (AW162) was able to selectively grow on mixed polystyrene, high-density polyethylene, and PET plastic monomers derived from metal-promoted oxidation and utilized both the aromatic and aliphatic substrates during growth. Similarly, the deletion of *pcaIJ* genes in strain AW162 (resulting in strain AW307) also enabled the production of PHA by cultivation in nitrogen-limited medium.^122^

Also inspired by the metabolic pathway present in *I. sakaiensis*, Sadler et al.^123^ developed an *in vivo* enzymatic pathway for the production of vanillin through the microbiological fermentation of TPA by an engineered *Escherichia coli* strain expressing enzymes comprising two pathways, divided in two plasmids. The first plasmid encoded TPADO and DCD dehydrogenase from *Comamonas* sp., while the second plasmid encoded a carboxylic acid reductase from *Nocardia iowensis* and a single-point mutant catechol *O*-methyltransferase with improved stereoselectivity from *Rattus norvegicus*. Initial PET hydrolysis was mediated by LCC, and authors reported up to 79% conversion of released TPA into vanillin through the biotechnological vanillin production pathway, equating 785 μM. One of the main limiting conditions for TPA conversion was cell permeability, as *E. coli* lacks a TPA transporter to import the substrate into the cytosol; the use of *n*-butanol and a pH condition of 5.5 were successful in improving the diffusion of TPA to the cellular interior. Furthermore, while LCC is indeed capable of releasing TPA directly, we hypothesize that the heterogeneous product solution arising from the slower hydrolysis of MHET by this enzyme could be remediated with the incorporation of corresponding MHET hydrolytic enzymes, potentially increasing final TPA and vanillin yields.

Besides the given examples, the usage of whole-cell biocatalysts engineered to express the metabolic enzymes necessary for the bioconversion of TPA has enabled the conversion of this substrate into added-value products such as gallic acid, pyrogallol, catechol, and muconic acid from PCA.^45,124^

Conversely, besides its application as a precursor of PET, EG has direct uses mainly as an antifreeze agent, but EG holds promise as an alternative carbon source or feedstock for the microbial synthesis of several products. For example, EG can undergo fermentation by *Gluconobacter oxidans* KCCM 40109 to yield glycolic acid, a common exfoliant with widespread use in the cosmetic industry.^124^

Like TPA, metabolic engineering efforts have also allowed the production of PHA from EG. Although *P. putida* KT2440 harbors the genetic framework necessary to metabolize EG, it is not able to naturally do it efficiently. Franden et al.^125^ elucidated the metabolic pathway to enable its growth on EG via the overexpression of glyoxylate carboligase (gcl) in combination with other genes in proximity transcribed as an operon (hydroxypyruvate isomerase (hyi), tartronate semialdehyde reductase (glxR), hydroxypyruavte reductase (ttuD), and pyruvate kinase (pykF)). The resulting strain (MFL185) showed increased growth rate and biomass yield on EG with little accumulation of glycolate and undetectable levels of glycolaldehyde. Furthermore, as a proof of concept, the engineered strain was cultured in nitrogen-limited conditions in M9 medium supplemented with 100 mM of EG as the only carbon source, and the authors confirmed the production of medium-chain-length PHA at a yield of 0.06 g of mcl-PHA per g of substrate.

EG can also be used as a carbon substrate for the production of aromatic chemicals. Using a previously engineered O_2_-tolerant *E. coli* variant (conferred by the introduction of two point mutations on 1,2-propanediol oxidoreductase (fucO)) with improved growth on EG and overexpression of glycolaldehyde dehydrogenase (aldA) for more efficient oxidation of EG to glycolate as a basis for further genetic modifications,^126^ Panda et al.^127^ deleted two genes (ΔtyrR and ΔpheA) to alleviate the repression on l *-*tyrosine production and stop l-phenylalanine (a byproduct) formation, respectively. Additional introduction of a plasmid overexpressing the feedback-resistant operon *aroG* and feedback-resistant *tyrA* allowed the efficient transformation of EG into l-tyrosine, outperforming the standard sugar feedstock glucose under the same optimized fermentation conditions. Furthermore, comparable titers of l-tyrosine were obtained using EG derived from acid hydrolysis of PET waste bottles to that yielded by the utilization of commercial EG.

## Summary and Outlook

PET hydrolytic enzymes can conventionally be classified as either true PET hydrolases or PET surface-modifying enzymes based on the extent of (bulk) polymer degradation they can achieve. In this classification, thermophilic cutinases comprise most of the former group, while the latter can still generally partially degrade PET. We hypothesize that these enzymes can find applications in PET biodegradation by further degrading released oligomeric molecules, remediating the heterogeneous products profile of PET hydrolases and increasing overall reaction efficiency by mitigating intermediate product inhibition, as well as enabling the recovery of the original building blocks of PET plastic.

The synergistic cooperation of complementary PET hydrolases and MHET/BHET hydrolases, whether through free enzyme systems or approaches like enzyme immobilization and fusion proteins, has demonstrated significant improvements in PET depolymerization efficiency. However, the development of thermostable enzyme variants and the refinement of dual-enzymes systems hold promise for creating more efficient and scalable would likely be necessary to keep these enzymes on par with thermophilic, highly effective PET hydrolases in terms of performance.

Furthermore, in the past decade, much effort has been directed to develop PET biodegradation systems. Quite a few start-up enterprises have been established, such as CARBIOS (France) and BreakPET (Mexico), providing the enzymatic degradation of PET as an alternative to conventional recycling methods. The elucidation of full enzymatic pathways for the assimilation of PET building blocks into bacterial metabolism has taken us a stage further, enabling the utilization of microorganisms to potentially generate sustainable chemicals and materials out of plastic waste via strategies such as metabolic engineering, synthetic biology, and precision fermentation.

Given the ubiquitous presence of plastic waste in the environment as a major marker of the Anthropocene, concern over the mismanagement of postconsumer plastic waste has grown rapidly. Recent developments in alternative recycling methods such as enzymatic biocatalysis are encouraging, but further research is still necessary. Upcycling of PET waste into high-value materials or substances is inspiring, and could bring a true circular, sustainable economy of PET that could very well bring revolutionary change.

## Supplementary Material



## Notes

Mutagenesis assays using *Is*MHETase (PDF)
