# Supplementary material for: It Is Dangerous to Go Alone: Strategies to Optimize PET Biocatalysis and Upcycling through Enzymatic Synergism
Source: ACS Omega. 2025 Oct 1;10(40):46218–37. doi: 10.1021/acsomega.5c02068 (PMC12529126; doi:10.1021/acsomega.5c02068)
Supplement: Supplementary file 1 [file ao5c02068_si_001.pdf]

Supporting Information

It's dangerous to go alone: strategies to optimize PET biocatalysis and upcycling through enzymatic synergism

Bruno Rampanelli Dahmer[a], Jeferson Camargo de Lima[a], José Fernando Ruggiero Bachega[b], Troy Wymore[c], and Luis Fernando Saraiva Macedo Timmers[a,d,*]

[a] Graduate Program in Biotechnology, Universidade do Vale do Taquari – Univates, Lajeado, RS, Brazil.

[b] Department of Pharmacosciences, Universidade Federal de Ciências da Saúde de Porto Alegre, Porto Alegre, RS, Brazil.

[c] Department of Chemistry, University of Pennsylvania, Philadephia, PA, USA.

[d] Graduate Program in Medical Sciences, Universidade do Vale do Taquari – Univates, Lajeado, RS, Brazil.

* To whom correspondence may be addressed: L. F. S. M. Timmers (Tel: +55 51 3714 7000 ext. 5860, Email: luis.timmers@univates.br)

Table S1. Mutagenesis assays with *Is*MHETase

Mutations	Reasoning	Reported findings
S225A, H528A, D492A	Confirmation of these residues as the catalytic triad.	Complete loss of activity towards MHET.[1,2]
H488A	Possible catalytic tetrad.	An unaltered turnover rate of MHET ruled out the existence of a catalytic tetrad.[2]
E226T	Oxyanion hole.	*Is*MHETase loses approximately 50% of its activity.[1]
S131G	Ser131 in *Is*MHETase is replaced by Gly in its two closest sequence homologues.	Results in poor affinity for MHET, K_M approximately 8-time higher, and reaction efficiency was reduced to about 3% of WT *Is*MHETase.[1]
W397A	Active site residue.	Increased enzymatic activity at high substrate concentrations at the expense of lower substrate affinity.[2]
F495A, I	Active site residue. *Is*MHETase presents a Phe495; in other tannase family members, this position is occupied by Ile.	Significantly decreases, or dramatically impairs, activity towards MHET.[1,2]
F415A, H, S	Active site residue. The positioning of F415 at the gate might be crucial for MHETase activity.	While F415A slightly lowers the enzymatic activity towards MHET, F415H significantly increases it.[2] F415S mutation also dramatically lowers activity towards MHET.[3]
S416G, A<br>S419G	Substrate interacting residues.	S416G showed slightly lower activity towards MHET. The S416A and S419G variants retain hydrolytic activity towards MHET and permit the conversion of BHET to TPA, likely due to the increased flexibility conferred to R411.[2]
F424Q, N, A, S, H, D, E, T, V, L, I	To introduce more potential hydrogen bond partners or to make the inner substrate-binding site larger by replacing F424 with several smaller residues.	F424Q and F424N significantly increase the turnover rate of BHET at the expense of significantly decreasing the turnover rate of MHET. Similarly, F424A and F424S also increase the turnover rate of BHET.[2] The variants investigated in the second study also showed somewhat decreased hydrolytic activity towards MHET, further demonstrating that these mutations have deleterious effects on the binding mechanism of this specific substrate. On the other hand, all these single point mutations resulted in 1.2 to 3.9-times increased activity towards BHET in comparison to WT *Is*MHETase.[3]
R411A, Q, K	Confirmation of the role of R411 in coordinating/guiding the binding orientation of the substrate. Subsequently, to evaluate whether the electrostatically less positive lysine is more favorable for stabilizing the ester bond between the TPA moiety and the inner EG moiety than R411A when BHET is used as a substrate.	Both R411A and R411Q almost completely abolish the conversion of MHET, but the removal of positive charges in these variants allow for a higher turnover rate of BHET (Palm et al., 2019). R411K retains over 40% of its relative activity towards MHET, while showing BHET hydrolysis activity 1.7-times higher than WT *Is*MHETase.[3]
R411A/S416A, R411A/S416G, R411A/S419G, R411Q/S416A, R411Q/S416G	Double mutants of previously characterized variants.	All double mutants were inactive towards MHET, but showed increased activity towards BHET, especially R411A/S416G.[2]
S416A/S419G, S416A/F424N, S419G/F424N		All three double mutants also increase activity towards BHET. Palm et al.[2] report a turnover rate of BHET of approximately 0.13

		s^{-1}. For the same mutation, Sagong et al.[3] report a 10.2-times increased activity towards the substrate.
F424N/H467N, F415H/F424N, F424N/L254N		These variants showed, in decreasing order, smaller improvements in enzymatic activity towards BHET compared to the other double mutants.[2]
R411K/F424N, R411K/F424V, R411K/F424I		The authors report that these variants showed higher MHET hydrolysis than the single point mutations, implying that the inclusion of R411K can increase activity towards this substrate. Furthermore, the R411K/F424V and R411K/F424I mutants were tested against a PET_5 oligomer, showing significantly enhanced activities compared to WT *Is*MHETase.[3]
R411A/S416G/S419G, R411A/S416G/F424N, R411A/S419G/F424N	Triple mutants of previously characterized variants.	All variants showed significantly increased activity and turnover rate towards BHET.[2]
R411K/S416A/F424N		Final triple variant developed by Sagong et al.[3] with high BHETase activity and enhanced hydrolytic capacity against PET film.
Lidless *Is*MHETase, Lidless/C224W/C529S, Lidless/C224H/C529S, and *Is*MHETase with 7 disulfide bonds	Given the structural similarities between the *Is*MHETase and *Is*PETase core domains, researchers were interested in understanding the role of the lid domain and the active site disulfide bond in *Is*MHETase on substrate specificity and MHET hydrolytic activity.	The active site disulfide bond residues (C224-C529) were mutated to W224 and S529 to match the active side of WT *Is*PETase, or to H224 and F529 to match the active site of a double mutant *Is*PETase previously shown to exhibit improved activity towards PET. Authors also designed a variant that included both the *Is*PETase-like disulfide bond (G489C/S530C) and the incorporation of a disulfide bond from *Ao*FaeB (S136C + grafted 15-residue loop that carries the partner cysteine).[1]<br>Very low activity against either MHET or PET was observed for any of the variants.[1]
T159V	Through combinatorial addition/reversion/recombination of consensus mutations, Saunders et al. introduced and combined several mutations to *Is*MHETase with the goal of increasing its soluble expression by facilitating protein folding.	T159V results in the loss of potential (weak) hydrogen bonds with the carbonyl oxygen of Ala140, but this potentially destabilizing effect appears to be mitigated by increased hydrophobic interactions.[4]
Y252F		Both Y252F and Y503W are located in a buried region of the protein structure and are spatially adjacent to one another. Although the Y503W mutation undoes a hydrogen bond to Gln236, the increased size of these sidechains results in better packing and hydrophobic interactions, which favors protein folding.[4]
Y503W		
T159V/Y252F/Y503W	Triple mutants of previously characterized variants.	MHETase-87 was the final triple mutant developed by Saunders et al.[4], exhibiting significantly increased soluble protein expression (>10-fold) and near-WT catalytic activity against MHET.
M192Y		MHETase-73 comprised the previous triple mutant plus the additional M192Y. The variant exhibited 14-fold improvement in whole-cell activity compared to WT-

		MHETase against the model substrate 1-naphtyl terephthalate. However, the addition of M192Y led to 5-fold lower activity of MHETase-73 on MHET, and its reversion generated an enzyme with comparable activity to WT *Is*MHETase.<br>The M192Y mutation results in the formation of a hydrogen bond with Glu226, which could be beneficial for protein folding but could affect the dynamics of the active site, as the latter residue is adjacent to the catalytic Ser225, evidenced by reduced K_M and k_{cat} values.[4]
**Suggested target mutations**		
Deletion of R229	To analyze the contribution of nearby residues to the activation energy of the rate-limiting step of the *Is*MHETase catalytic mechanism in QC/MM molecular dynamics simulations.	High increase in activation energy (+3.6 kcal/mol) suggests that this residue is important in stabilizing E226 and the oxyanion hole.[5]
Deletion of E230		Decreases the energy barrier (-2.3 kcal/mol), meaning that this residue could be a target for substitution.[5]
Deletion of H488 or its substitution with a nonpolar residue		Consistent with the findings of Palm et al. (2019)[2] the mutation could maintain or slightly increase the turnover rate of *Is*MHETase. The deletion of H488 decreases the activation energy (-2.4 kcal/mol).[5]
Deletion of A493		Significantly decreases the activation energy (-3.3 kcal/mol).[38]
Deletion of S491		Appears to be relevant in stabilizing the transition state, as it is in close proximity to H528 and its deletion increases the energy barrier (+2.2 kcal/mol).[5]
S136, T179, P186, D446, D449, W453	Using molecular dynamics simulations and Markov State Models to compare the conformational features between active and inactive states of *Is*MHETase, the authors propose mutation hotspots that could destabilize the inactive states of the enzyme without influencing the active states.	It is suggested that these hydrogen bonds have higher hydrogen bond occupancy in the inactive states of the enzyme than in its active ones. Weakening these interactions could increase the distribution of active states of *Is*MHETase, potentially affecting substrate binding affinity.[6]

REFERENCES

(1) Knott, B. C.; Erickson, E.; Allen, M. D.; Gado, J. E.; Graham, R.; Kearns, F. L.; Pardo, I.; Topuzlu, E.; Anderson, J. J.; Austin, H. P.; Dominick, G.; Johnson, C. W.; Rorrer, N. A.; Szostkiewicz, C. J.; Copié, V.; Payne, C. M.; Woodcock, H. L.; Donohoe, B. S.; Beckham, G. T.; McGeehan, J. E. Characterization and Engineering of a Two-Enzyme System for Plastics Depolymerization. *Proc. Natl. Acad. Sci.* **2020**, *117* (41), 25476–25485. https://doi.org/10.1073/pnas.2006753117.

(2) Palm, G. J.; Reisky, L.; Böttcher, D.; Müller, H.; Michels, E. A. P.; Walczak, M. C.; Berndt, L.; Weiss, M. S.; Bornscheuer, U. T.; Weber, G. Structure of the Plastic-Degrading Ideonella Sakaiensis MHETase Bound to a Substrate. *Nat. Commun.* **2019**, *10* (1), 1717. https://doi.org/10.1038/s41467-019-09326-3.

(3) Sagong, H.-Y.; Seo, H.; Kim, T.; Son, H. F.; Joo, S.; Lee, S. H.; Kim, S.; Woo, J.-S.; Hwang, S. Y.; Kim, K.-J. Decomposition of the PET Film by MHETase Using Exo-PETase Function. *ACS Catal.* **2020**, *10* (8), 4805–4812. https://doi.org/10.1021/acscatal.9b05604.
(4) Saunders, J. W.; Damry, A. M.; Vongsouthi, V.; Spence, M. A.; Frkic, R. L.; Gomez, C.; Yates, P. A.; Matthews, D. S.; Tokuriki, N.; McLeod, M. D.; Jackson, C. J. Increasing the Soluble Expression and Whole-Cell Activity of the Plastic-Degrading Enzyme MHETase through Consensus Design. *Biochemistry* **2024**, *63* (13), 1663–1673. https://doi.org/10.1021/acs.biochem.4c00165.
(5) Pinto, A. V.; Ferreira, P.; Neves, R. P. P.; Fernandes, P. A.; Ramos, M. J.; Magalhães, A. L. Reaction Mechanism of MHETase, a PET Degrading Enzyme. *ACS Catal.* **2021**, *11* (16), 10416–10428. https://doi.org/10.1021/acscatal.1c02444.
(6) Peng, X.; Lu, C.; Pang, J.; Liu, Z.; Lu, D. A Distal Regulatory Strategy of Enzymes: From Local to Global Conformational Dynamics. *Phys. Chem. Chem. Phys.* **2021**, *23* (39), 22451–22465. https://doi.org/10.1039/D1CP01519B.
